# CRISPR-Cas System, a Possible “Savior” of Rice Threatened by Climate Change: An Updated Review

**DOI:** 10.1186/s12284-023-00652-1

**Published:** 2023-09-09

**Authors:** Nabeel Shaheen, Shakeel Ahmad, Salem S Alghamdi, Hafiz Mamoon Rehman, Muhammad Arshad Javed, Javaria Tabassum, Gaoneng Shao

**Affiliations:** 1 Seed Center and Plant Genetic Resources Bank, Ministry of Environment, Water & Agriculture, Riyadh, 14712 Saudi Arabia; 2 Plant Production Department, College of Food and Agriculture Sciences, King Saud University, Riyadh, Saudi Arabia; 3 Centre for Agricultural Biochemistry and Biotechnology (CABB), University of Agriculture Faisalabad, Faisalabad, 38000 Pakistan; 4 Department of Plant Breeding and Genetics, Faculty of Agricultural Sciences, University of the Punjab, Lahore, 54590 Pakistan; 5 State Key Laboratory of Rice Biology and China National Center for Rice Improvement, National Rice Research Institute, 310006 Hangzhou, China; 6 Zhejiang Lab, 310006 Hangzhou, China

**Keywords:** *Oryza sativa L*., Gene editing, CRISPR-Cas system, Climate-resilience, Food security

## Abstract

Climate change has significantly affected agriculture production, particularly the rice crop that is consumed by almost half of the world’s population and contributes significantly to global food security. Rice is vulnerable to several abiotic and biotic stresses such as drought, heat, salinity, heavy metals, rice blast, and bacterial blight that cause huge yield losses in rice, thus threatening food security worldwide. In this regard, several plant breeding and biotechnological techniques have been used to raise such rice varieties that could tackle climate changes. Nowadays, gene editing (GE) technology has revolutionized crop improvement. Among GE technology, CRISPR/Cas (Clustered Regularly Interspaced Short Palindromic Repeats/CRISPR-associated protein) system has emerged as one of the most convenient, robust, cost-effective, and less labor-intensive system due to which it has got more popularity among plant researchers, especially rice breeders and geneticists. Since 2013 (the year of first application of CRISPR/Cas-based GE system in rice), several trait-specific climate-resilient rice lines have been developed using CRISPR/Cas-based GE tools. Earlier, several reports have been published confirming the successful application of GE tools for rice improvement. However, this review particularly aims to provide an updated and well-synthesized brief discussion based on the recent studies (from 2020 to present) on the applications of GE tools, particularly CRISPR-based systems for developing CRISPR rice to tackle the current alarming situation of climate change, worldwide. Moreover, potential limitations and technical bottlenecks in the development of CRISPR rice, and prospects are also discussed.

## Introduction

Food security is being challenged by several factors including but not limited to climate change. It is one of the biggest challenges being faced by humanity not only in this era but can also probe the future generations, if not resolved. It is estimated that a huge chunk of world’s population does not have access to enough food for their survival for years, and about two million people are currently facing nutrient deficiency, leading to their stunted growth, as well (Ahmad et al. 2021a, b, c). In such a critical situation, rice (*Oryza sativa L*.), being one of the major food crops and a staple food of almost half of the world’s population, can play a pivotal role in global food security and can also be a key solution provider. However, about 90% of its cultivation is happening in Asian countries, which seems more vulnerable to changing climatic conditions. The *japonica* and *indica* sub-species of rice are dominant in the rice cultivation system, however, *indica* rice holds more share in rice market (Uyeh et al. 2021). It is estimated that the world’s population can rise to 8.5 billion by 2030, and about 25% increase in rice production would be required to meet the global food by then (Ahmad et al. 2021a, b, c). Whereas climate changes such as severe drought, heat, salinity, cold, deviation in precipitation pattern, increasing diseases and insect pest attacks are posing serious effects on both rice yield and its nutritional quality. For instance, it is projected that the temperature would rise by 8℃, the average drought index will be 129, which is 52.45 now, and the sea level could also rise by 2100 that may cause food havoc (Li et al. 2009). Moreover, every degree increase in temperature could increase 3% and 7% average precipitation and humidity, respectively, that would promote diseases and insect attacks on the crops (Rezvi et al. 2022). Consequently, the world may suffer with impaired food production system, huge yield losses, and ultimately global food insecurity.

Rice is highly sensitive to climate change as it requires optimum irrigation and a suitable temperature to grow normally. It is reported that 1℃ temperature increase could decrease paddy yield by up to 3.44%. While the same increase in precipitation could decrease 0.12% and 0.21% yield and crop harvest, respectively (Alam et al. 2011). So, as a matter of concern, it is reported that the Malaysian granary area’s temperature could increase from 0.3℃ to 0.5℃ along with increased precipitation from 133 to 200 mm (Firdaus et al. 2020). Ancha (2012) has also described that the Cambodian climate could face a 2.5℃ and 8.3% increase in temperature and rainfall, respectively, which would lead to humid environments in rainy seasons and lower humidity in dry seasons. Therefore, swiftly changing climatic scenario has become a central concern for the whole agricultural production system, especially rice, to ensure current and future global food security. Thus, being a major food crop, new climate smart rice cultivars should be developed that could withstand changing climatic conditions to feed the ever-growing human population and to end hunger, globally (Arunrat et al. 2020; Hussain and Ahmad 2022; Riaz et al. 2022).

In this regard, classical plant breeding has not only contributed in the past but also played a crucial role in current rice breeding programs for its improvement (Fiaz et al. 2021; Patel et al. 2020). Whereas the utilization of conventional plant breeding techniques (CPBTs) for crop improvements is limiting with the development of new plant breeding technologies (NPBTs), which are more efficient, robust, cheaper, and less time-consuming to produce promising results as compared to CPBTs. In addition, modern problems like rapid climate change, soil degradation, pollutant accumulation, and altered rainfall pattern also restrict the application of CPBTs due to their low efficiency and slow pace (Ahmad et al. 2021a, b, c). By keeping in view, the recent environmental challenges and high food demand, NPBTs especially gene editing (GE) could be an effective alternative to the CPBTs that can help to end hunger and ensure food security, globally (Ahmad et al. 2020, 2021a, b, c). Among different GE systems CRISPR-Cas (Clustered Regularly Interspaced Palindromic Repeats-CRISPR-associated Protein) system provides a robust, efficient, and economical mode of action that can alter the genetic makeup of crop plants without introducing foreign DNA, a major controversy in the acceptance of genetically modified organisms (GMOs) developed through transgenic breeding method (Ahmad et al. 2021a, b, c; Monsur et al. 2020). Additionally, the current availability of genome sequences and information of vast number of plant species has made it a more suitable tool to manipulate any specific gene/s for the creation of new desirable crop varieties.

**Fig. 1:**
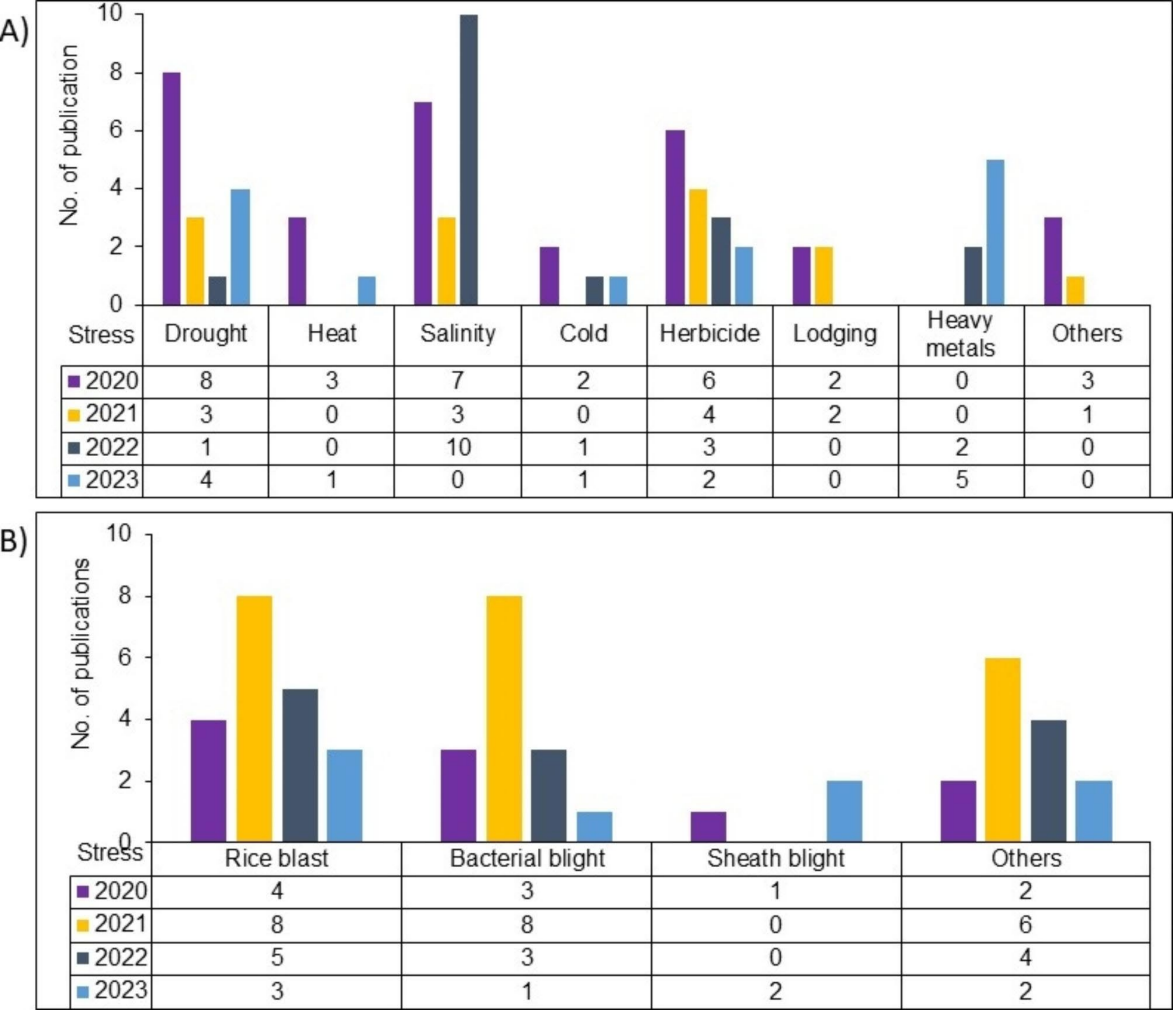
Record of research focusing on the development of abiotic (**A**) and biotic (**B**) stress tolerant rice lines using CRISPR-Cas system between 2020 - To date

Here, to give an updated picture of the application of CRISPR-Cas systems in rice from 2020 to Present (“Present” refers to March 2023), we have explored five different scholarly databases including Google Scholar (http://scholar.google.com), Pubmed (https://pubmed.ncbi.nlm.nih.gov/), Scopus (https://www.scopus.com/home.uri?zone=header&origin=), DOAJ (Directory of Open Access Journals) (https://doaj.org/) and Refseek (https://www.refseek.com/) and collected the relevant literature using web filters and various keywords i.e., rice, *Oryza sativa* L., gene editing, CRISPR, genome editing in rice, climate change, biotic resistance, and abiotic tolerance. Finally, fully screened, and curated data have been presented and discussed below in this article to highlight the potential of CRISPR-Cas based GE system in rice improvement (Fig. 1).

Although several reviews on GE in rice have been published previously (Fiaz et al. 2019; Khan et al. 2021; Mishra et al. 2018; Tabassum et al. 2021), but an up-to-date progress of rice GE was needed due to its high value and significant contribution to food security. Thus, this spatiotemporal mini review primarily aims to summarize the recent success stories and progress of CRISPR-based GE systems, especially CRISPR-Cas9, over the last three years in the creation of climate-smart rice lines. Moreover, regulatory aspects and future directions are also discussed in detail.

## Can CRISPR Help to Breed New Climate-resilient Transgene-free Rice Varieties?

**Fig. 2:**
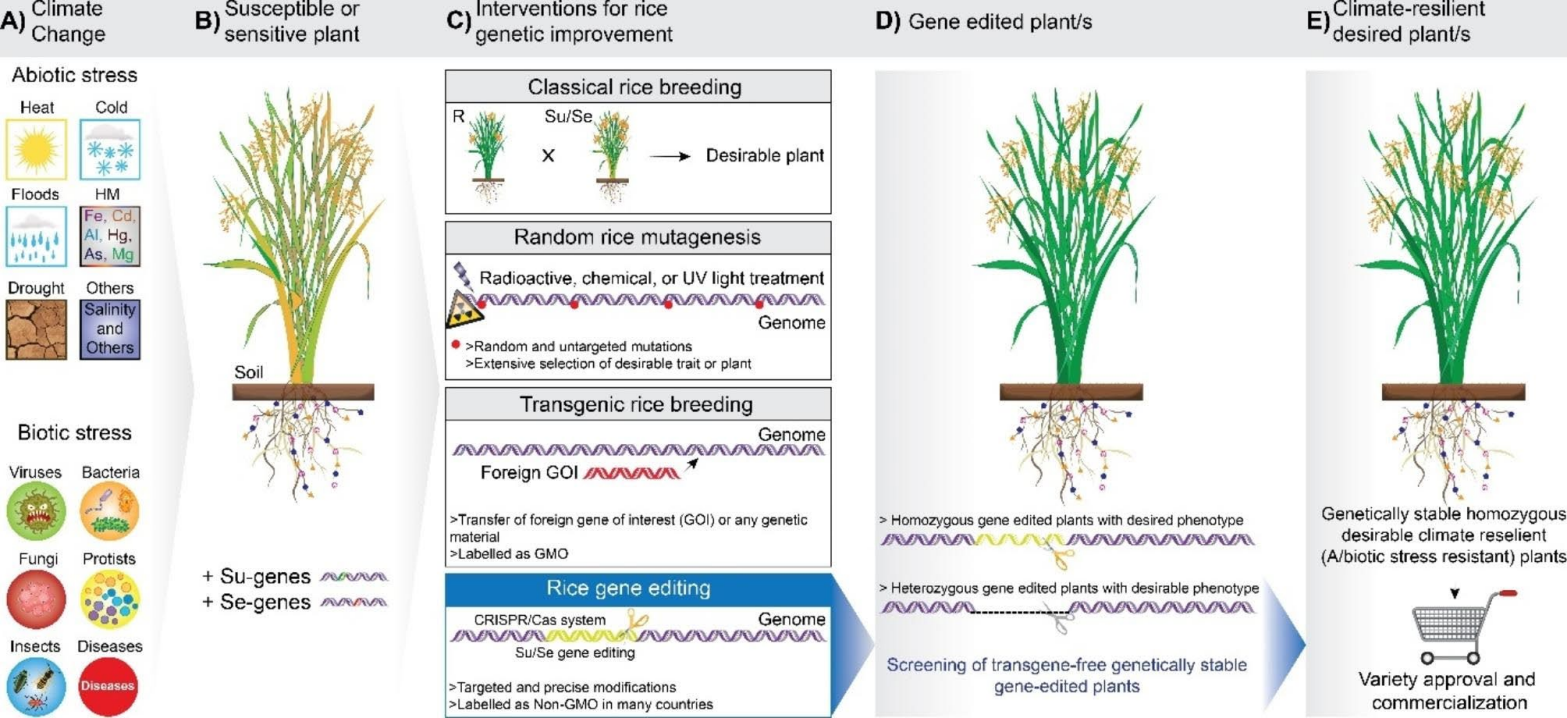
Illustration of production of publicly acceptable transgene-free climate resilient rice varieties using gene editing tools. **(A)** Different biotic and abiotic stresses. **(B)** Diseased or under stress (A/biotic) plants having susceptibility (Su) and sensitivity (Se) genes. **(C)** Various crop improvement breeding interventions. **(D)** Gene-edited plant/s (T0) developed using GE tools e.g., CRISPR-Cas-based GE system. At this stage plant/s could have heterozygous or homozygous mutation, hence screening of homozygous plants is required. **(E)** Final genetically stable homozygous transgene-free desired rice plant/s that can be released commercially as a potential new variety or can be used in rice breeding programs

Nowadays, CRISPR-Cas based GE is more valued due to its robustness, accuracy, and applicability in crop plants, especially in rice. Compared to other GE techniques, CRISPR-Cas solely can play a significant role in the development of climate resilient rice lines by performing multiple tasks including but not limited to knock-out, knock-in, epigenetic changes and transcriptional regulation of different genes controlling various traits (Ahmad et al. 2021a, b, c). Further, CRISPR-Cas system based indels, homologous recombination based targeted sequence alteration, and base pair changes that could not be differentiated from natural mutation are spared from GMOs regulation in several countries (Turnbull et al. 2021). Consequently, CRISPR edited foreign DNA free rice lines will be used in rice breeding programs for developing new rice varieties that could be commercialized (Fig. 2). For instance, according to this spatiotemporal study, CRISPR-Cas based GE systems have been used in total 126 research projects since 2020 that have been conducted on the theme of development of climate resilient transgene-free rice lines or validation of the role of gene/s involved in any biotic or abiotic stress tolerance. Out of these published research, 74 and 52 research found related to abiotic and biotic stresses, respectively (Fig. 1). Hence these publications have been further explored and reviewed critically, below in this section, to confirm the promising role of CRISPR technology in developing abiotic (drought, heat, cold, herbicide, salinity, lodging, heavy metal, and others) and biotic stress (bacterial leaf blight, rice blast, sheath blight, and others) tolerant rice varieties.

### Re-wiring of Rice Genome via CRISPR for Developing Abiotic Stress Tolerance

Abiotic stresses, particularly drought, heat, cold, salinity, herbicide, and salinity cause substantial yield losses in rice by damaging its growth and developmental processes. It is the need of the hour to develop such rice varieties that could withstand rapidly changing abiotic environmental conditions. CRISPR-Cas systems have been proved as a promising tool in this regard (Table 1). Hence, this section covers its recent applications against abiotic stresses in rice.

**Table 1:** Summary of applications of CRISPR-Cas based gene editing systems against abiotic stresses in rice between 2020–2023

Stress Name	Gene/s Targeted	Gene nature/Gene function	Gene editing system/gRNA used	Results	Reference
Drought	*OsPYL9*	Abscisic acid receptor gene	CRISPR-Cas9/two gRNAs	Higher ABA accumulation and lower stomatal conductance	(Usman et al. 2020)
	*OsmiR818b*	Drought gene	CRIPSR-Cas9/ single gRNA	Lower resistance	(Chung et al. 2020)
	*OsmiR535*	Modulating cold stress response/negative regulator of cold stress	CRISPR-Cas9/ single gRNAs	Enhanced tolerance against cold, salinity, and drought stresses	(Yue et al. 2020)
	*OsABA8ox2*	an ABA catabolic gene	CRISPR-Cas9/ single gRNA	Improved drought tolerance	(Zhang et al. 2020a, b)
	*OsAAA-1* and *OsAAA-2*	Drought sensitive genes	CRISPR-Cas9/ four gRNA	Enhanced drought tolerance along with improved grain yield	(Lu et al. 2020)
	*OsSDD1* and *OsRSD1*	Clustering stomata	CRISPR-Cas9/two gRNAs	decreased stomatal density	(Yu et al. 2020)
	*OsBC1L1* or *OsBC1L8*	Stomatal patterning and production	CRISPR-Cas9	stomatal clustering and stomatal production	(Li et al. 2021a, b)
	*OsEPFL10 and STOMAGEN*	Negative regulators	CRISPR-Cas9/ single gRNA	Increased tolerance	(Karavolias et al. 2021)
	*OsMADS26*	Transcription factor	CRISPR-Cas9/three gRNAs	Proof of concept	(Anjala and Augustine 2022)
	*OsSAPK3*	Osmotic stress protein kinases	CRISPR-Cas9/ single gRNA	Reduced sensitivity to drought	(Lou et al. 2023)
	*OsIPA1*	Encodes transcriptional factor	CRISPR-Cas9/ single gRNA	Improved drought tolerance by regulating *SNAC1*	(Chen et al. 2023a, b)
	*OsLKP2*	Cuticular wax biosynthesis	CRISPR-Cas9	Enhanced tolerance	(Shim et al. 2023)
	*OsWRKY76*	Transcription factor	CRISPR-Cas9	Weak drought tolerance	(Zhang et al. 2023a, b)
Heat	*OsNTL3*	Negative regulator	CRISPR-Cas9/ single gRNA	Heat sensitivity	(Liu et al. 2020a, b, c)
	*OsLRK1*	regulator of leaf-based dark respiration	CRISPR-Cas9/ single gRNA	Compromised growth at 35 ℃	(Qu et al. 2020)
	*OsSRL10*	Regulates thermotolerance and leaf morphology	CRISPR-Cas9/ single gRNA	Sensitivity to high temperature	(Zhang et al. 2023a, b)
Salinity	*OsGTγ-2* (TF)	Positive regulator of salinity response in rice	CRISPR-Cas9/single gRNA	Salinity hypersensitive plants	(Liu et al. 2020a, b, c)
	*Osgs3* and *Osdep1* heterotrimeric G proteins	Do signal transmission	CRISPR-Cas9/single gRNA	Improved salinity tolerance	(Cui et al. 2020)
	*OsMPT3;1 and OsMPT3;2*	Mitochondrial phosphate transporter genes	CRISPR-Cas9/single gRNA	Increased Na+/K + ratio	(Huang et al. 2020a, b, c)
	*OsPQT3*	Negative regulator	CRISPR-Cas9/two gRNAs	Enhanced resistance with improved grain yield	(Alfatih et al. 2020)
	*OsSERK2*	co-receptor in brassinosteroids signaling	CRISPR-Cas9/two gRNAs	Salinity sensitivity	(Dong et al. 2020)
	*OsqSOR1*	root gravitropic responses	CRISPR-Cas9/single gRNA	Reduced the stress in saline paddy fields	(Kitomi et al. 2020)
	*OsSST*	regulating the release of root exudates	CRISPR-Cas9/single gRNA	Better plant adaptation under saline conditions	(Lian et al. 2020)
	*OsRR22*	Involved in salt tolerance in rice	CRISPR-Cas9	Higher salt tolerance	(Tripathy et al. 2021)
	*OsMKK1, OsMKK6*, and *OsMKK1/6*	Affect lateral root growth	CRISPR-Cas9/ two gRNAs	Increased auxin contents and enhanced lateral roots growth under salinity	(Yang et al. 2021)
	*OsHKT2;1*	Involved in mineral transportation	CRISPR-Cas9/ single gRNA	Enhanced salt tolerant phenotypes by reducing sodium ion and ROS accumulation.	(Wei et al. 2021a, b)
	*OsbHLH024* (TF)	Involved in plant growth and stress response	CRISPR-Cas9/ single gRNA	Improved salt tolerance	(Alam et al. 2022a, b, c)
	*OsRR22*	Involved in salt tolerance in rice	CRISPR-Cas9/ single gRNA	Improved salt tolerance	(Han et al. 2022)
	*OsVDE*	Negatively regulator of salinity stress	CRISPR-Cas9/ single gRNA	Higher survival rate and stomatal conductance	(Wang et al. 2022a, b, c)
	*OsbHLH024*	Transcription factor	CRISPR-Cas9/ two gRNAs	Enhanced salt tolerance	(Alam et al. 2022a, b, c)
	*OsBEAR1*	Regulator of salt response	CRISPR-Cas9 and artificial miRNA	Mutation by both methods enhanced salt sensitivity	(Teng et al. 2022)
	*OsbHLH044*	Transcription factor	CRISPR-Cas9/ two gRNAs	Salinity sensitivity	(Alam et al. 2022a, b, c)
	*OsXLG2* and *OsXLG4*	extra-large GTP-binding protein	CRISPR-Cas9/ single gRNA	Double mutant exhibited salinity tolerance	(Biswal et al. 2022)
	*OsBadh2*	Related to synthesis of aromatic component	CRISPR-Cas9/ single gRNA	Improved tolerance in non-aromatic variety (Huaidao#5)	(Prodhan et al. 2022)
	*OsGLYI3*	Sensitive gene to salinity	CRISPR-Cas9/ single gRNA	Sensitivity to salinity stress	(Liu et al. 2022)
Cold	*OsGL1-11*	Wax synthesis	CRISPR-Cas9	Sensitivity to salinity stress	(Zhao et al. 2022)
	*OsPIN5b, OsGS3, OsMYB30*	Cold sensitive	CRISPR-Cas9/ two gRNAs	Improved cold tolerance with higher yield	(Zeng et al. 2020a, b)
	*OsAnn5*	Positive regulator of cold stress	CRISPR-Cas9/ single gRNA	Cold sensitivity	(Que et al. 2020)
	*OsTCD3*	Cold induced gene	CRISPR-Cas9/ single gRNA	Cold sensitivity	(Lin et al. 2020)
	*OsWRKY76*	Sensitive gene	CRISPR-Cas9	Decreased cold tolerance	(Zhang et al. 2022a, b)
	*OsCOLD11*	Positive regulator	CRISPR-Cas9 / single gRNA	Reduce chilling tolerance	(Li et al. 2023)
Herbicide	*OsHIS1*	Sensitive gene	CRISPR-Cas9 cytidine deaminase fusion/ four gRNAs	Herbicide sensitive	(Komatsu et al. 2020)
	*OsALS*	Primary target for Bispyribac sodium herbicide	CRISPR-Cas9/ single gRNA	Herbicide tolerant	(Butt et al. 2020)
	*OsALS1*	Primary target for Bispyribac sodium herbicide	CRISPR-Cas base editor/ 63 gRNAs	Herbicide tolerant	(Kuang et al. 2020)
	*OsALS*	Primary target for Bispyribac sodium herbicide	CRISPR-Cas12a gene targeting/two crRNAs	Efficient biallelic gene targeting	(Li et al. 2020a, b)
	*OsALS*	Primary target for Bispyribac sodium herbicide	CRISPR-Cas9/ single gRNA	novel allele G628W after after transversion of G to T in *OsALS* gene regiom conferred improved herbicide tolerance	(Wang et al. 2021a, b, c, d)
	*OsALS*	Tolerant to bispyribac herbicide	Base editing at four different locations	Improve herbicide tolerance	(Zhang et al. 2021a, b)
	*OsAFB4*	Auxin receptor	CRISPR-Cas9/ two gRNAs	Improved resistance to picloram	(Guo et al. 2021)
	*OsACC1*	Negative regulator	prime-editing-library-mediated saturation mutagenesis	Herbicide resistant	(Xu et al. 2021a, b, c, d)
	*OsPUT1/2/3*	Transporter gene	CRISPR-Cas9/ single gRNA	paraquat resistance	(Lyu et al. 2022)
	*OsCYP76C6*	Cytochrome encoding gene (isoproturon herbicide)	CRISPR-Cas9/ single gRNA	Increased conjugates and isoproturon metabolites	(Zhai et al. 2022)
	*OsALS*	Primary target of multiple herbicides	CRISPR-Cas9/ single gRNA	Strong resistance	(Liying et al. 2022)
	*OsEPSPS*	Herbicide gene	Prime editing	Herbicide resistance	(Butt et al. 2020)
	*OsALS*	Primary target of multiple herbicides	CRISPR-Cas9	Herbicide tolerance	(Zafar et al. 2023)
	*OsHPPD3*	4-hydroxyphenylpyruvatedioxygenase inhibitor	CRISPR-Cas12a	Herbicide tolerance	(Wu et al. 2023)
Heavy metals	*OsNRAMP5*	Member of transporter gene family	CRISPR-Cas9/ single gRNA	Decreases in root uptake of Pb	(Chang et al. 2022)
	*OsPMEI12*	Do modification of methyl esterification	CRISPR-Cas9/ single gRNA	Better growth under phytohormone stress and increased fresh and dry weight under cd stree	(Li et al. 2022a, b, c)
	*OsLCD*	Involved in Cd accumulation	CRISPR-Cas9/ single gRNA	Reduce Cd translocation and enhanced Cd tolerance	(Elkonin et al. 2023)
	*OsACE2*	Encodes an acetyltransferase	CRISPR-Cas9	Detoxification to oxyfluorfen	(Xu et al. 2023)
	*OsmiR535*	Fine-tuning regulator of genes	CRISPR-Cas9	Tolerance to Cd toxification	(Yue et al. 2023)
	*OsNramp5*	Member of transporter gene family	CRISPR-Cas9/ single gRNA	Manganese, copper, and selenium decreased in knock out lines	(Feng et al. 2023)
	*OsCERK1*	Negative regulator	CRISPR-Cas9	Tolerance to cupric oxide nanoparticles	(Chen et al. 2023a, b)
Lodging	*OsGW2*	Primarily controls grain weight	CRISPR-Cas9/ single gRNA	Lodging resistance	(Yamaguchi et al. 2020)
	*OsRhoGDI2*	Plant height	CRISPR-Cas9/ single gRNA	Semi dwarf	(Wang et al. 2020a, b, c, d)
	*OsSd1*	Involved in plant height	CRISPR-Cas9/ two gRNAs	Lodging resistance	(Wang et al. 2022a, b, c)
	*OsSd-1*	Dwarfing gene	CRISPR-Cas9/ two gRNAs	Lodging resistance	(Beyene et al. 2022)
Drought and salinity	*OsDST*	Negative regulator	CRISPR-Cas9/ two gRNAs	Higher tolerance level against salinity and moderate against osmotic stress	(Santosh Kumar et al. 2020)
	*OsbZIP72*	Transcription Factor	CRISPR-Cas9/ single gRNA	Sensitivity to drought and salinity	(Baoxiang et al. 2021)
	*OsNPF8.1*	Positive regulator	CRISPR-Cas9	Less tolerance in knock out lines	(Diyang et al. 2023)
Drought and heat	*OsNAC006*	Transcription Factor	CRISPR-Cas9/ single gRNA	Drought and heat sensitivity	(Wang et al. 2020a, b, c, d)
drought and abscisic acid	*OsERA1*	Negative regulator	CRISPR-Cas9/ single gRNA	Hypersensitivity to ABA and enhanced drought tolerance	(Ogata et al. 2020)
	*OsAFP1*	Negative regulator	CRISPR-Cas9	Decreased water loss and ABA sensitivity	(Tianshun et al. 2021)
Others	*OsDOFs* (TFs)	Plant specific transcription factors	CRISPR-Cas9/ gRNA	11 DOFs regulated heading date under long day conditions and 9 DOFs controlled heading date under short day conditions.	(Huang et al. 2020a, b, c)
	*OsLPR3*	Involved in response to phosphate	CRISPR-Cas9/ single gRNA	Improved Pi tolerance	(Lin et al. 2023)
	*OsIRO3*	Repressor to Fe homeostasis	CRISPR-Cas9/ single gRNA	Accumulation of Mg, Fe, and ROS increased in leaves	(Wang et al. 2020a, b, c, d)
	*OsSAW1*	Positive regulator	CRISPR-Cas9/ single gRNA	Male sterile plants	(Wang et al. 2020a, b, c, d)
	*OsHAK8*	Involved in K^+^ uptake	CRISPR-Cas9/ single gRNA	Reduced K^+^uptake	(Wang et al. 2021a, b, c, d)

#### Drought

Drought stress is one of the major threats that limits crop productivity and can affect food security significantly (Zhang et al. 2023a, b). It is projected that about half of the global cultivated land would suffer from severe water scarcity by the end of 2050 (Kamanga et al. 2018; Mushtaq et al. 2018). To overcome the possible upcoming consequences, production of drought tolerant crop varieties, especially rice, is undeniable. In this regard, researchers have utilized the CRISPR-Cas based GE systems to knock out the undesirable sensitivity (Se) causing gene/s and have created new drought tolerant rice lines (Table 1).

For instance, 366 bp deletion in *drought and salinity tolerant 1* gene through CRISPR-Cas9 enhanced water retention against dehydration stress followed by broader leaves production (Santosh Kumar et al. 2020). The phenotype of CRISPR-edited mutant plants showed tolerance against drought stress. Similarly, a CRISPR-Cas9 based null mutation in *pyrabactin resistance-like* (*OsPYL9*) gene resulted in higher cuticular leaf wax accumulation, lower transpiration rate, and increased grain weight along with drought tolerance (Usman et al. 2020). Chung et al. (2020) also identified 66 drought induced microRNAs and of these *OsmiR535* and *OsmiR818b* were found to be drought responsive. The 5 bp- homozygous deletion in drought responsive *OsmiR535* induced tolerance against PEG, dehydration, NaCl and abscisic acid (ABA) stresses (Yue et al. 2020). Likewise, the loss of function of *OsABA8ox2* gene, an ABA 8,-hydroxylase encoding gene, generated via CRISPR-Cas9 system produced a long and narrow rooting system that is beneficial to acquire water under drought conditions (Zhang et al. 2020a, b). In addition, stomatal density largely affects water transpiration thus playing a critical role in drought stress. For instance, the CRISPR-Cas9 based *stomata developmental defect 1* (*osrsd1*) mutants had decreased stomatal density that prevented water loss under dehydration stress. Consequently, mutant lines also exhibited altered expression of other stomatal related genes i.e., *stomatal density and distribution 1* (*OsSDD1*), and showed tolerance against drought stress (Yu et al. 2020). Lu et al. (2020) identified two drought-sensitive genes (*OsAAA-1* and *OsAAA-2*) through screening of the activation tagging population. Thus, CRISPR-Cas9 mediated disruption introduced pre-mature truncation of protein that resulted in increased grain yield under limited water availability. Moreover, disruption of *epidermal patterning factor* 10 (*EPFL10*) through CRISPR-Cas9 system caused lower stomatal density followed by improved water conservation compared to the wild (Karavolias et al. 2021). Similarly, CRISPR-Cas9 generated *osmotic stress/ABA–activated protein kinases* (*sapk3-1* and *sapk3-2*) mutants displayed lower water loss and normal plant growth under drought conditions (Lou et al. 2023). Cuticular wax synthesis under drought stress inhibits water loss and its production could prevent the plant from severe damages. Under drought stress, both knockdown and knockout mutants of *lov kelch repeat protein 2* (*Oslkrp2*) gene made 10% more cuticular wax along with increased leaf size, which resultant improve the level of tolerance of plants against drought conditions (Shim et al. 2023). In contrast to these studies, scientists have also identified some positive regulators (genes) of drought stress whose disruption has brought drought sensitivity. These genes include *nitrate transporter 1/peptide transporter family* (*OsNPF8.1*) (Diyang et al. 2023), and *ideal plant architecture 1* (*IPA1*) genes (Chen et al. 2023a, b). Finally, it could be concluded that CRISPR-Cas mediated over-expression of these genes could contribute to produce drought tolerant rice lines. Similarly, numerous stress-related factors (TFs) have also been found to be positively associated with drought tolerance. For example, CRISPR-Cas9 mediated *NAM, ATAF1/2*, and *CUC2* (*OsNAC006*) knock-out lines impaired morpho-physiological traits together with decreased antioxidants (catalase, peroxidase dismutase, and peroxidase) growth following drought stress (Wang et al. 2020a, b, c, d). Similarly, CRISPR-Cas 9 oriented disruption of *OsWRKY76* (w76-1 and w76-2) TF has brought severe symptoms of wilting, electrolyte leakage, water loss, and chlorosis after treating with 20% PEG (Zhang et al. 2023a, b). Additionally, the rice genome also has 89 basic region/leucine zipper motif (*bZIP*) TFs that are involved in regulating numerous regulatory and stress related pathways. Two base pairs deletion in the first codon of *OsbZIP72* TF led to the premature termination and phenotype of this mutation caused withering of seedlings against drought compared to over-expressed lines demonstrating better morphological appearance against drought (Baoxiang et al. 2021).

#### Heat

According to temperature stats recently published by FAO in May 2023, 1.4 °C was the average annual temperature change in the world in 2022 (FAO 2023). However, it is predicted that the average global temperature would increase up to 2℃ to 4℃ by the end of 21st century, and this increase in temperature could significantly affect crop productivity (Jagadish et al. 2015). The global mean temperature could rise to 3℃ by the mid twenty-first century (Field et al. 2012). Hence, it is becoming more alarming as it is reported that 1℃ increase in mean temperature could affect 6–7% grain yield of crop plants (Lesk et al. 2016). Considering the adverse effects of prolonged heat stress scientists have made multiple efforts using CRISPR-based GE system to produce heat resilient rice lines (Table 1).

For example, *OsNAC006* TF is regulated by indole acetic acid, gibberellin, H_2_O_2_, ABA, cold, heat, PEG, and NaCl treatments. CRISPR-Cas9 mediated null mutants of *OsNAC006* have displayed thermo-tolerance in rice plants followed by elevated H_2_O_2_, O^2-^ levels (Wang et al. 2020a, b, c, d). Similarly, CRISPR-Cas9 oriented disruption of *NTL* TF at two sites produced n*tl3-1* and *ntl3-2* mutants. The n*tl3-1* produced truncated protein whereas *ntl3-2* mutant exhibited a new C-terminus in addition to a truncated protein. Interestingly, regardless of post GE protein structure both mutants showed thermo-tolerance at 45℃ for 5 days with lower survival rate (Liu et al. 2020a, b, c). On the other hand, there are also some genes that are positively associated with heat tolerance whose expression is essential to ensure heat tolerance. For example, *leucine rich repeat receptor kinase* (*LRK1*) is positively correlated with leaf dark respiration and CRISPR-Cas9 based single bp mutation at the 508th nucleotide resulted in premature termination of protein by introducing the stop codon at the 180th amino acid sequence. Exposure of *lrk1* mutants to 35℃ for 10 days reduced dark respiration along with retarded morphological growth (Qu et al. 2020). Likewise, three mutations consisting of 1-bp, 2-bp, and 4-bp deletion in the first exon of *semi rolled leaf 10* (*SRL10*) gene through CRISPR-Cas9 generated three independent mutant lines that exhibited semi rolled leaf phenotype and compromised thermo-sensitivity (Zhang et al. 2023a, b). Consequently, the application of CRISPR-Cas9 system confirms that constitutive expression of these genes is needed to induce thermotolerance in rice.

#### Salinity

Salinity stress greatly affects the seedling and reproductive stages of rice plants by introducing osmotic, oxidative, and ion toxicity stresses. Interestingly, salt tolerant rice is a preferred grain crop to utilize the saline-alkali and coastal tidal lands, which has great utilization potential (Han et al. 2022). Numerous studies have been executed to find out and to characterize the salinity associated gene (s) through CRISPR-Cas mediated GE.

For instance, *soil surface rooting 1* (*OsqSOR1*) is a homolog to *deep rooting 1* (*AtDRO1*) gene that governs shallow root growth angle. One bp substitution in the 3rd exon of *OsqSOR1* gene caused premature truncation and produced soil surface root which improves salinity tolerance (Kitomi et al. 2020). Likely, double mutants of *extra-large GTP-binding protein* (*osxlg1*/*osxlg4*) genes also increased root length that led to improved salinity tolerance (Biswal et al. 2022). Further, CRISPR-Cas9 oriented two mutations viz., -20 bp in M16 exon and − 1 bp in M18 exon of *OsRR22* gave higher root and shoot weight and a greater number of fresh leaves against 0.8% NaCl treatment (Han et al. 2022). Heterotrimeric G proteins are involved in regulating the stress responses in plants. CRISPR-Cas9 based null mutations in G protein encoding genes (*gs3*, and *dep1*) induced salinity tolerance (Cui et al. 2020). Similarly, the *paraquat tolerance 3* (*OsPQT3*) gene could switch off the stress mechanism through an off-switch mechanism and disruption of the first and second exons of *OsPQT3* gene resulted in higher salt tolerance (150mM NaCl) together with improved germination (Alfatih et al. 2020). For salinity tolerance, balance between Na + and other salts is crucial in salinity tolerance and sensitivity. in this regard, Lian et al. (2020) have observed lower K + concentration and improved morphological growth in *open stomata 2* (*ossst*) mutants against 150mM NaCl. Like genes, TFs could also perform compartmentation of Na + and K + to induce salt tolerance and a single bp deletion in *OsbHLH024* TF improved oxidative stress tolerance by maintaining the balanced level of Ca2+, Zn2+, and Mg2 + minerals (Alam et al. 2022a, b, c). Moreover, steroid hormones also have their role in stress tolerance, but they are difficult to use in plant improvement due to their complex role. Dong et al. (2020) have disrupted *SERK2*, one of the steroid component genes that enhanced salinity tolerance. Similarly, ABA biosynthesis also has some roles in salinity tolerance and CRISPR-Cas9 based disruption of *violaxanthin de-epoxidase* (*OsVDE*) gene has brought salinity tolerance followed by increased ABA level higher survival rate, and stomatal closure (Wang et al. 2022a, b, c). Although gene disruption could contribute to stress tolerance, genotypic background largely affects the induction of stress tolerance. In this regard Prodhan et al. (2022) have disrupted the *betaine aldehyde dehydrogenase* (*OsBadh2*) gene with CRISPR-Cas9 simultaneously in Jiahua#1 (WT_JH) and Huaidao#5 (WT_HD) cultivars. Surprisingly mutation in *OsBadh2* gene induced salinity tolerance in WT_HD lines only, indicating the significant effect of the genetic background to acquire the stress tolerance.

Conversely, positive regulators of salinity tolerance have also been reported whose disruption introduced such characteristics that have produced salt sensitive rice lines. For instance, disruption of *Ca2 + sensor, calmodulin* (*OsCaM1*) gene produced lower primary root, and weakened lateral root length with reduced root density confirming its sensitivity to salt stress (Yang et al. 2021). Likewise, CRISPR-Cas9 based mutagenesis in the first exon of *glyoxalase* (*OsGLYi3*) gene yielded saline hypersensitivity following elevated methylglyoxal, and glyoxalase I activity (Liu et al. 2022). Balance of Na + and K + in root zones greatly affects salinity tolerance in crop plants. Identically, knock-out lines of *mitochondrial phosphate transporters* (*OsMPT3*;*1* and *OsMPT3*;*2*) genes exhibited compromised morphological growth and reduced Na + efflux and K + and Ca + influx as well (Huang et al. 2020a, b, c). Like genes, TF could also positively regulate the salinity tolerance. CRISPR-Cas9 oriented disruption of a trihelix (*OsGTγ-2*) TF produced salt hypersensitive phenotypes followed by imbalanced K + and Na + accumulation (Liu et al. 2020a, b, c). Likewise, CRISPR-Cas9 mediated editing of 3rd exon of BEAR1, a *bHLH* TF, produced salt sensitive phenotypes i.e., reduced plant height compared to control (Teng et al. 2022). Interestingly, knocked out mutants of *bHLH044* TF also produced salt sensitive lines. However, this sensitivity was due to the elevated reactive oxygen species levels followed by higher lipid peroxidation and H_2_O_2_ levels (Alam et al. 2022a, b, c).

#### Cold

Rice is vulnerable to chilling stress and induction of chilling tolerance is pivotal to expanding its cultivation to northern areas characterized by much lower annual temperatures (Li et al. 2023). Chilling tolerance is a complex trait and is controlled by several quantitative trait loci such as COLD1, qCTS12/GSTZ2, CTB4a, and Ctb1 (Liu et al. 2018). Therefore, several attempts have been made to induce chilling tolerance or to investigate genes involved in chilling tolerance. Many tolerance causing genes have been reported in the past (Le et al. 2022; Romero and Gatica-Arias 2019), and latest are presented here (Table 1). Such as, CRISPR-Cas9 mediated disruption of a *cold tolerance gene* (*OsMYB30*), *panicle length gene* (*OsPIN5b*), and *grain size gene (GS3*) genes yielded cold tolerance along with improved yield. Additionally, two triple mutants comprised of *ospin5b*/*gs3*/*osmyb30*-*25* and *ospin5b*/*gs3*/*osmyb30*-*4* also exhibited improved cold tolerance together with enhanced grain yield (Zeng et al. 2020a, b). Likewise, exposure of CRISPR-Cas9 based *osatp4* and *ostcd3* mutants to lower temperature possessed improved cold tolerance coupled with decreased chlorophyll contents (Lin et al. 2020). Moreover, *WRKY* TFs are also associated with cold tolerance. For instance, CRISPR-Cas9 oriented disruption of *OSWRKY63* TF introduced cold tolerance in rice (Li et al. 2021a, b). In contrast, like other stresses, positive regulators of cold stress have also been reported. Like, editing of the fifth exon of *annexin* (*OsAnn5*) gene caused cold sensitivity in rice seedlings (Que et al. 2020). Further, CRISPR-Cas9 based editing of *OsWRKY76* TF produced cold sensitive rice lines (Zhang et al. 2022a, b). Though editing of positive regulators is not required but the application of CRISPR-Cas9 system helped in elucidating their confirm role in cold stress. Hence it proves that CRISPR-Cas9 system does not only help in developing new cold stress tolerant germplasm but also playing its critical role in functional analysis of the newly identified and cloned gene/s.

#### Herbicide

Weeds are another big challenge to rice and hinder its production significantly. Several efforts, including but not limited to the utilization of conventional, mechanical, and chemical approaches, have been made to cope with this problem so far. Nevertheless, these strategies have been proven expensive, less efficient, and/or hazardous to health and ecosystem (Chauhan 2013; Kuang et al. 2020; Wu et al. 2023). The development of herbicide tolerant genotypes using CRISPR-Cas systems are the need of the hour and could be one of the popular strategies to overcome the challenge of weeds to rice and to improve its production, globally. For that, extensive studies have been conducted in last three years to investigate the possible roles of several genes towards herbicide tolerance and their utilization in the development of herbicide tolerant rice lines through CRISPR-Cas systems (Table 1).

*Acetolectase synthase* (*ALS)* gene, an herbicide resistant gene catalyzes the first step of biosynthesis of branched amino acids that are the first target of Bispyribac sodium (BS) and other herbicides. Recently, conversion of tryptophan to leucine at 548th position of *ALS* gene improved BS tolerance in basmati rice (Zafar et al. 2023). Likewise, Liying et al. (2022) have also edited *OsALS* genes earlier and obtained three types of mutants viz., *ALSS627N* and *1884G-A*, *ALSS627N*, and *ALSS627N*/*G628E* that were resistant to imidazole ethylinicotinic acid. Moreover, auxin hormones play a variety of roles in plants including embryogenesis, tissue elongation, and vascular differentiation. Dicot weeds are controlled by auxinic herbicides. CRISPR-Cas9 directed disruption of auxin signaling f-box (*OsAFB4*) auxin receptor induced tolerance against picloram and 2,4-dichlrophenoxyacetic acid (Guo et al. 2021). Moreover, single bp substitution has proved their crucial role in producing herbicide tolerant rice lines. Xu et al. (2021a, b, c, d) have developed prime-editing-library-mediated saturation mutagenesis to increase the substitution rate at the target site (s) and have identified 16 diverse mutations in *OsACC1* corresponding to herbicide resistance. Additionally, paraquat herbicide resistance has also been achieved in CRISPR-Cas9 based triple mutants of *OsPUT* knock-out lines (Alfatih et al. 2020). Beside BS, glyphosate is a widely used herbicide, but it also affects the main crop. A single bp substitution of 96G to A in glyphosate resistant gene (*EPSPS*) induced herbicide tolerance (Jiang et al. 2022). Hence, in this case it can also be concluded that CRISPR-based base editors have not only improved the rice resistance against herbicides but also proved that it can generate mutants that could be mimicking natural mutants. Besides, unlike drought, salinity and heat stresses, positive regulators of herbicide tolerance have not been reported in the last three years.

#### Heavy Metal

Heavy metals are undesirable for plants and humans too as their exposure to the human body could damage organs even at lower concentration. Heavy metals are non**-**biodegradable, stable, and persistent that made them hazardous for human consumption. Cadmium (Cd) is one of the most threatening heavy metals for living organisms and its pollution in agricultural soil has continuously been increasing (Zhao et al. 2015). Cd is not only affecting rice yield and quality, but it is also a serious threat to human health as well. Cd interacts with metabolic processes and could cause various cancers and bone diseases in the human body (Clemens 2019). Although numerous studies have been conducted to find out the Cd associated genes but unfortunately, till now only a few genes have been found that can take part in Cd metabolism. Such as, null mutants of *low cadmium* (*oslcd*) gene accumulated less Cd in shoots under the influence of Cd-contaminated soil (Elkonin et al. 2023). Similarly, the knocked-out mutants of *mir535* of brown rice accumulated 35% less Cd compared to the control under controlled stress of 2 µmol/L Cd (Yue et al. 2023). Moreover, Chang et al. (2022) have also got Cd tolerance with limited manganese accumulation by disrupting *natural resistance associated macrophage protein* (*OsNramp5*) gene with CRISPR-Cas9. Besides Cd, copper oxide nanoparticles (CuO NPs) are also creating toxication in rice plants and *oscerk1* mutants had CuO NPs tolerance followed by the increased accumulation and regulation of H_2_O_2_ and antioxidant system respectively (Chen et al. 2023a, b). In addition to heavy metal contamination, pesticides residuals are another source of contaminated cultivated soils. Xu et al. (2023) have identified *acetyltransferase* (*OsACE2*) gene that has a primary role in catabolizing the oxyfluorfen pesticide residuals in soil. However, CRISPR-Cas9 based downregulation of this gene resulted in compromised morphological growth and higher oxyfluorfen accumulation suggesting its positive association with oxyfluorfen catabolization.

#### Others

Besides the prominent stresses, other minor stresses have also been addressed through CRISPR systems. These stresses include Fe homeostasis, lodging, effects of short and long-day span on heading, Pi (phosphorous) starvation, and K^+^ (potassium ion) uptake. For detail overview please refer to Table 1.

### Re-wiring of Rice Genome via CRISPR System for Developing Resistance Against Biotic Stresses

Biotic stresses including diseases and insects cause up to 40% (and sometimes complete) yield losses in rice. Rice is attacked by many pathogens including bacterial, fungal, and viral, which cause several kinds of diseases in rice that eventually hamper or stop the growth and development processes in rice. Hence, developing pathogen and insect resistant rice varieties are quite important to achieve global food security. In this regard, the CRISPR-Cas system is contributing significantly to developing biotic stress resistant rice lines (Table 2). So, the contributions of CRISPR-Cas system are highlighted below in this section.

**Table 2:** Summary of last 3-year applications of CRISPR-Cas mediated gene editing systems against biotic stresses in rice

Disease/Biotic Stress	Targeted gene/s	Gene nature	Gene editing system/gRNA used	Results	Reference
Rice blast	*OsBsr*-*d1*	Regulation of redox stage of plant cells	CRISPR-Cas9/single gRNA	Enhanced resistance against rice blast	(Zhu et al. 2020)
	*OsFLR1 and OsFLR13*	Involved in rice-*Magnaporthe oryzae* intercation	CRISPR-Cas9/16 gRNAs	Increased susceptibility	(Huang et al. 2020a, b, c)
	*OsFLR2 and OsFLR11*			Enhanced resistance	
	*OsFLR1*			Enhanced pathogen attack	
	*OsFLR2*			Delayed pathogen attack	
	*OsDjA2* and *OsERF104*	Susceptibility genes	CRISPR-Cas9/two gRNAs	Improved resistance	(Távora 2021)
	*OsUGT74J1*	SA-glucosyltransferases	CRISPR-Cas9/two gRNAs	Enhanced Salicylic Acid accumulation and resistance against RB	(Tezuka et al. 2021)
	*OsWRKY93*	Transcription factor	CRISPR-Cas9/ single gRNA	Sensitivity to pathogen	(Li et al. 2021a, b)
	*OsGRF18*	Positive regulator	CRISPR-Cas9/ single gRNA	Improved resistance	(Yudong et al. 2021)
	*OsUBC26*	a rice ubiquitin-conjugating enzyme	CRISPR-Cas9	Compromised resistance	(Liu et al. 2021a, b, c)
	*OsXa7*	Broad spectrum resistance gene	CRISPR-Cas9/two gRNAs	Sensitivity to pathogen	(Chen et al. 2021)
	*OsECBS*	Produce casbane-type diterpenoids	CRISPR-Cas9/ RNAs	Impaired resistance	(Liang et al. 2021)
	*OsPita-Fuhui2663*	Negative regulator	CRISPR-Cas9/ single RNA	Resistance	(He et al. 2022)
	*OsPib*	R gene	CRISPR-Cas9/ single RNA	Displayed more symptoms after pathogen inoculation	(Xie et al. 2022)
	*OsWRKY7*	Transcription factor	CRISPR-Cas9/ single RNA	Sensitivity to blast fungus	(Tun et al. 2023)
Rice blast	*Ospi-ta*	Susceptibility gene	Intron-targeted insertion strategy of CRISPR-Cas9/single gRNA	Disease resistance with improved agronomic characteristics	(Xu et al. 2020)
	*OsPi21*	Broad spectrum resistance gene	CRISPR-Cas9/two gRNAs	Resistance without affecting agronomic traits	(Nawaz et al. 2020)
	*OsPiPR1*	Partial R gene	CRISPR-Cas9/single gRNA	Partially reduced blast resistance	(Liu et al. 2020a, b, c)
	*OsDjA2* and *OsERF104*	Susceptibility genes	CRISPR-Cas9/single gRNA	Improved resistance	(MILAzzo et al. 2022)
	*OsDjA2* and *OsERF104*	Susceptibility genes	CRISPR-Cas9/single gRNA	Resistance	(Távora et al. 2022)
	*OsHRC*	Negative regulator	CRISPR-Cas9	Resistance	(Ding et al. 2023)
Bacterial blight	*OsXa13* promoter	Sensitive gene	CRISPR-Cas9/two gRNAs	Disease resistance	(Li et al. 2020a, b)
	*OsSWEET14*	Susceptibility gene	CRISPR-Cas9/three gRNAs	Resistance against bacterial blight	(Zafar et al. 2020)
	*OsSWEET14*	Susceptibility gene	CRISPR-Cas9/two gRNAs	Strong resistance with improve plant height	(Zeng et al. 2020a, b)
	*OsEBEAvrXa23*	Susceptible gene	CRISPR-Cas9 knock-in	Resistant	(Wei et al. 2021a, b)
	*OsTFIIAγ1* or *OsTFIIAγ5* (TF)	Involved in pathogen interaction	CRISPR-Cas9/ two gRNAs	Resistance	(Xu et al. 2021a, b, c, d)
	*OsSWEET14*	Susceptibility gene	CRISPR-Cas9/single gRNA	Moderate to high resistance	(Arulganesh et al. 2021)
	*OsSWEET14*	Susceptibility gene	CRISPR-Cas9/single gRNA	Improved resistance	(Duy et al. 2021)
	*OsUPT* box	Promoter region	CRISPR-Cas12a/ single crRNA	Improved resistance	(Yu et al. 2021)
	*OsSWEET14*	Susceptibility gene	CRISPR-Cas9/single gRNA	Less susceptibility	(Kim et al. 2021)
	*OsPrx30*	Bacterial blight responsive gene	CRISPR-Cas9/single gRNA	Increased resistance	(Liu et al. 2021a, b, c)
	*OsSWEET13*	Susceptibility gene	CRISPR-Cas9/single gRNA	Could interfere with binding site of pathogen effector binding element	(Diana et al. 2022)
	*OsXa13*	Recessive pleiotropic gene	CRISPR-Cas9/ two gRNA	Disease resistance	(Li et al. 2022a, b, c)
	*OsCPK24*	Positive regulator	CRISPR-Cas9/single gRNA	More susceptibility to BLB	(Lu et al. 2023)
Sheath blight	*OsPP2A-1*	Positive regulator	CRISPR-Cas9/single gRNA	Susceptible	Lin et al. (2021)
Rice blast and bacterial blight	*OsPi21, OsBsr-d1*, and *OsXa5*	Susceptibility genes	CRISPR-Cas9/single gRNA	Resistance	(Tao et al. 2021)
Bacterial blight and leaf streak	*OsSWEET11, OsSWEET14* and *OsSULTR3;6*	Susceptibility genes	CRISPR-Cas9/single gRNA	Resistance	(Ni et al. 2021)
	*OsBsr-d1, OsPi21 and OsERF922*	Resistant genes	CRISPR-Cas9/single gRNA	Improved resistance in both single and triplet mutants	(Zhou et al. 2022)
Rice tungro disease	*OseIF4G*	Required to translate viral RNA viruses	CRISPR-Cas9/single gRNA	Enhanced tolerance	(Nithya et al. 2020)
Rice black-streaked dwarf virus	*OseIF4G*	Required to translate viral RNA viruses	CRISPR-Cas9/single gRNA	Enhanced tolerance	(Wang et al. 2021a, b, c, d)
	*OsAGO2*	Negative regulator	transposon-insertion or CRISPR/Cas9	Over-expression enhanced susceptibility and mutant showed resistance	(Wang et al. 2021a, b, c, d)
Southern rice black-streaked dwarf virus	*OsARF17* (TF)	Resistance gene	CRISPR-Cas9/single gRNA	Increased susceptibility	(Zhang et al. 2020a, b)
	*Osv-ATPase d*	Mediating virus resistance	CRISPR-Cas9/single gRNA	Resistance	(Lu et al. 2021)
Bacterial leaf streak	*OsSULRT3; 6*	Susceptibility gene	CRISPR-Cas9/single gRNA	Resistance	(Xu et al. 2021a, b, c, d)
Brown plant hopper	*OsAOC and OsMYC2*	Involved in signaling	CRISPR-Cas9/single gRNA	Pathogen performed better on knock out lines	(Xu et al. 2021a, b, c, d)
	*OsCKXs*	Negative regulator	CRISPR-Cas9	Enhanced resistance	(Zhang et al. 2022a, b)
*Meloidogyne graminicola*	*OsBet v1*	Involved in defense against microbial pathogens	CRISPR-Cas9/ two gRNAs	Increased susceptibility	(Li et al. 2022a, b, c)
Rice blast and bacterial blight	*OsMORE1a*	Immunity related gene	CRISPR-Cas9/single gRNA	Resistance to *M. oryzae* and *Xanthomonas oryzae pv. oryzae* but increased susceptibility to *Cochliobolus miyabeanus*,	(Kim et al. 2022)
Rice blast, bacterial blight and sheath blight	*OsUMP1*	natural allele of proteasome maturation factor	CRISPR-Cas9	Broad spectrum resistance to *Magnaporthe oryzae*, *Rhizoctonia solani*, *Ustilaginoidea virens* and *Xanthomonas oryzae pv. oryzae*	(Hu et al. 2023)
Rice black-streaked dwarf virus disease and southern rice black-streaked dwarf virus disease	*OsAP47*	Negatively regulates the resistance	CRISPR-Cas9/ two gRNAs	Enhances resistance against both stresses	(Wang et al. 2022a, b, c)
Yellow mottle virus	*OsCPR5.1*	Nucleoporin paralogs	CRISPR-Cas9/ two gRNAs	Disruption resulted in RYMV resistance	(Arra et al. 2023)
	*OsCPR5.2*	Nucleoporin paralogs	CRISPR-Cas9/ two gRNAs	Susceptibility after disruption	

#### Rice Blast

Rice blast is a fungal disease caused by *Magnaporthe oryzae*; a hemi biotrophic fungus. It has been extensively studied due to its lethal damage to rice. Scientists have also developed a Patho-system of rice *M. oryzae* which has been used as a primary model to study plant-microbe interaction (Li et al. 2019). Using this model, scientists have conducted multiple studies in the last three years to produce rice blast resistant lines through CRISPR-Cas systems.

In this regard, Huang et al. (2020a, b, c) have edited the N terminal sequences of four *Ferrona like receptor* (*FLR*) genes that are involved in rice *M. oryzae* interaction. Double knocked out lines of *FLR2* and *FLR11* receptors depicted enhanced resistance, while single knocked out lines of *FLR1* and *FLR13* showed susceptibility indicating their negative and positive association respectively with blast resistance. Similarly, single mutant lines of *FLR1* and single knock out lines of *FLR2* receptor delayed the pathogen infection respectively. Differences in resistance/susceptibility could be due to the altered expression of other genes. Likewise, MILAzzo et al. (2022) have achieved blast resistance characterized by lower disease symptoms following the disruption of *OsDjA2* and *ethylene-responsive factor104* (*ERF104*) genes independently. Salicylic acid (SA) may have a crucial role in the plant immune system and its indirect association with blast resistance has also been reported. Downregulation of *UGT74J1*, a *UDP-glucosyltransferase* gene induced several PR-related genes along with improved blast resistance and higher SA accumulation. However, confirmation of this hypothesis needs further investigation whether blast resistance is due to the overexpression of PR-related genes or higher SA accumulation (Tezuka et al. 2021). Further, impairing avirulence activity of pathogen has also been proven to be fruitful to induce blast resistance. For example, CRISPR-Cas9 based *ubiquitin-conjugating enzyme26* (*osubc26*) mutants depicted impaired avirulence activity by impairing proteasome through degradation of AvrPiz-t cells Liu et al. (2021a, b, c). Addition to gene disruption, CRISPR-Cas based gene knock-in approach has also been used to induce blast resistance in rice lines. Xu et al. (2020) have induced blight resistance by introducing exon #2 of *Pi*-*ta* gene (blast resistant gene) into *pi-ta* (susceptible gene) gene resulting in rice blast resistance.

In contrast, there are also such genes and TFs have been reported in the last three years whose expression is required to ensure the blast resistance. For example, CRISPR-Cas9 based null mutations of *OsPib* (Xie et al. 2022), *ent-casbene synthase* (Liang et al. 2021), *peroxidase3* (*OsPerox3*) (Liu et al. 2021a, b, c), *growth-regulating factor4* (*OsGRF4*) (Yudong et al. 2021), and *partial resistance1* gene (*OsPiPR1*) (Liu et al. 2020a, b, c) genes have increased blast susceptibility. Similarly, some TFs particularly WRKY TF gene family have been found to be positively associated with blast resistance and disruption of *WRKY45, OsWRKY93* and *OsWRKY7* TFs caused more susceptibility (Li et al. 2021a, b; Tun et al. 2023). Finally, the expression of specific genes may conclude to ensure blast resistance.

#### Bacterial Blight

Bacterial blight of rice caused by *Xanthomonas oryzae* pv. *Oryzae* (*Xoo*), is a destructive rice disease that can reduce the rice grain yield by up to 75%. *Xoo* could activate the host susceptibility gene followed by seizing the host machinery through its endogenous transcription activator-like effectors (TALEs). Moreover, TALEs could bind to effector binding elements (EBE) to cause bacterial diseases that are present in the upstream regions of *SWEET* genes or other plant susceptible genes. Targeted mutation in the promoter of *OsSWEET14* gene characterized with AvrXa7 deletion exhibited improved resistance against *Xoo*. Consistently Zeng et al. (2020a, b) have targeted the 1st and 3rd exons of *Sugars Will Eventually be Exported Transporter14* (*OsSWEET14*) gene and have obtained resistance against the Asian and African races of *Xoo* (AXO1947). In addition to gene knock-out, scientists have also engineered other *SWEET* genes as well and Diana et al. (2022) have introduced indels with CRISPR-Cas9 in the promoter of susceptibility gene (*OsSWEET13*). However, phenotypic evaluation has yet to be performed. Like *SWEET* genes, pathogen’s disease inducing genes including *Xa13*, *Xa1*, and *Xa23* have also been subjected to GE to explore their role towards rice blight resistance. It is worth noticing here that these two genes could contribute to broad spectrum resistance (BSR) as wellWei et al. (2021a, b) have reported BSR to blight by knocking of *EBEAvrXa23* elements into the promoter regions of susceptible allele (*xa23*). Furthermore, altering EBEs of *Xa13* gene’s promoters also resulted in blight resistance (Li et al. 2020a, b). Besides EBEs, UPT boxes are also of great importance in acquiring blight resistance. CRISPR-Cas12a mediated site-specific mutations in UPT box of *Xa13* gene gave blight resistant phenotypes followed by disturbed TALEs binding sites (Yu et al. 2021). Hence, CRISPR-based GE tools can be some promising tools to combat bacterial blight and reduce yield losses being happening due to it.

#### Sheath Blight

Sheath blight is caused by *Rhizoctonia solani* and causes withering and lodging of the entire plant that could reduce grain yield by up to 50%. Identification of susceptibility genes is a prerequisite to induce disease resistance in crop plants. In this regard numerous efforts have been made to identify and to edit the disease responsive genes through CRISPR-Cas system in last three years. In contrast to other studies, most genes are positively associated with sheath blight resistance. For example, employing of CRISPR-Cas9 system on *Protein phosphate* (*OsPP2A1*) gene yielded five mutants; *pp2a-1-1* had 1 and 2 bp insertions in 1st exon, and *pp2a-2* had 1 bp deletion in 11th exon. Moreover, *pp2a-3*, and *pp2a-4*, had 1 bp insertion and 2 bp insertion in their 1st and 2nd exon respectively, whereas *pp2a-5* contained 1pb deletion in the 1st exon too. Interestingly, all these mutants depicted hypersensitivity to sheath blight disease compared to control (Lin et al. 2021). Similarly, Jung and colleagues have found that NH4^+^ uptake is positively associated with sheath blight disease as *osamt1* lines (an ammonium transporter) exhibited hypersensitivity against *R. solani* isolates (Jung et al. 2023).

#### Others

Rice genome editing has also been attempted to induce other biotic stress resilience as well including microbial organisms, brown plant hopper (Zhang et al. 2022a, b), rice tungro virus (Kumam et al. 2022), rice black streaked dwarf virus (Wang et al. 2021a, b, c, d; Wang et al. 2022a; Wang et al. 2022b; Wang et al. 2022c), southern rice black streaked dwarf virus (Lu et al. 2021; Wang et al. 2022a, b, c) and yellow mottle virus (Arra et al. 2023). In addition to these, some genes have also shown resistance to multiple pathogens or diseases (Table 2). Hence, all these applications show that CRISPR-based GE tool has great potential to develop climate-smart rice varieties that can help us to achieve food security, globally.

## Challenges, Opportunities, and Future Perspective

Several GE tools, including restriction enzymes, zinc finger nucleases, Transcription activator-like effector nucleases, CRISPR-Cas systems, and transposases have been used for creating a better rice for a long time. However, there is no doubt that among these, CRISPR-Cas based GE systems (especially CRISPR-Cas9 system) have been the most adopted GE tool due to its higher accuracy, robustness, ability to modify the target sequence, and applicability to maximum number of crop plants without any restrictions. Several improvements have been observed in rice DNA and RNA editing using CRISPR-Cas systems since its first application in rice in 2013. The world has witnessed the successful application of different versions of CRISPR-Cas system, prominently CRISPR-Cas9/-Cas12/-Cas13, DNA/RNA base editors, and prime editors, in developing new desirable rice lines (Aqib et al. 2022; Malzahn et al. 2019; Tang et al. 2018; Zhang et al. 2021a, b). Besides its huge potential over others, CRISPR-Cas systems also have some limitations and challenges, including but not limited to off-target effects, bigger size of Cas proteins, limited protospacer adjacent motif (PAM) sites as they are important to determine the target site (e.g., in case of Cas protein “NGG“ is the PAM site), efficiency and accuracy of targeting the desired DNA/RNA fragment, and/or production of transgene free CRISPR-edited mutants (Ahmad et al. 2020). To overcome these limitations, many efforts have been made and many more are needed.

For example, multiple alternative PAM sites (including NAG, NGA, NNGG, NNG, NAA etc.) have been identified to improve the efficiency of the system. The wild-type Cas9 protein can efficiently identify the NGG and NAG PAM sites in rice. Moreover, according to Meng et al. (2023) using NGA alone or in combination with NAG could enhance editing efficiency. In contrast, it is reported that despite of first recognition of NAG PAM by SpyCas9, NGG has shown strong affinity for SpyCas9 protein (Kleinstiver et al. 2015). Hence, the identification of new orthologs of Cas9 nuclease or engineering of available Cas9 are needed that could identify and show strong affinity with new and efficient PAM sites in the genome. Otherwise, the identification of the target/s in the genome for gene disruption using CRISPR-Cas systems could be PAM independent (Collias and Beisel 2021). Moreover, the identification of new variants of Cas9 nuclease can also solve the problem of the transformation of bigger size SpyCas9 nuclease into plants e.g., a smaller Cas9s nuclease has recently been engineered and used effectively in therapeutics as an alternative (Schmidt et al. 2021).

Off-target effects are another growing challenge that should be addressed. Off-targets could be of two types especially in case of base editors: (1) Cas protein dependent, or (2) Cas protein independent. Hence, both types are crucial and need ultimate solution. Nowadays, several *in silico*, in vitro, *in vitro in cellulo*, and *in cellulo* based methods are available for identifying genome-wide CRISPR-Cas off-target sites (Tao et al. 2023). Recent advancements in genomics and next-generation sequencing (NGS) would certainly help to minimize the off-targeting and to identify the CRISPR edited plants without off-target mutations. Recently, Amit et al. (2021) have also developed a model based on NGS data to reduce the off targets. Moreover, the development of base editing, prime editing and CRISPR-Cpf1 type systems have made CRISPR-based GE technology more reliable due to their minimal off-target activities. Collectively, it can be predicted that off-target effects can be further reduced from minimal to even zero level.

Besides off-targets, tissue culture is one of the key steps involved in delivering CRISPR/Cas system reagents into target plant. Optimization of tissue culture for each targeted crop and various economically important tree species (which are difficult to propagate and/or near to their extinction) is a major bottleneck on their way to improvement using CRISPR-Cas based GE technology. In this regard, quite a few successes and advancements have been observed in recent days. Fortunately, researchers have recently combined grafting and mobile CRISPR to avoid the laborious and time-consuming tissue culture technique and delivered the CRISPR/Cas9 reagents as RNA from transgenic roots (rootstock) to distal parts of unmodified grafted scion, where it is translated into proteins to induce heritable mutagenesis at desired loci (Yang et al. 2023). This technique has been critically reviewed by (Zaman et al. 2023), and it is demonstrated that it has tremendous potential to produce transgene-free and genetically stable plants not only in field and vegetable crops, but also fruits and other economical trees. Similarly, Cao et al. (2023) have also developed an extremely simple cut–dip–budding (CDB) tissue culture free delivery system, which uses *Agrobacterium rhizogene* to inoculate explants and does not need sterile conditions. It has been demonstrated that CDB is a useful tool to achieve the heritable transformation of plant species in multiple plant families. In addition, several in planta transformation attempts have been made to perform tissue culture free GE (Hamada et al. 2018; Liu et al. 2021a, b, c) .

**Fig. 3:**
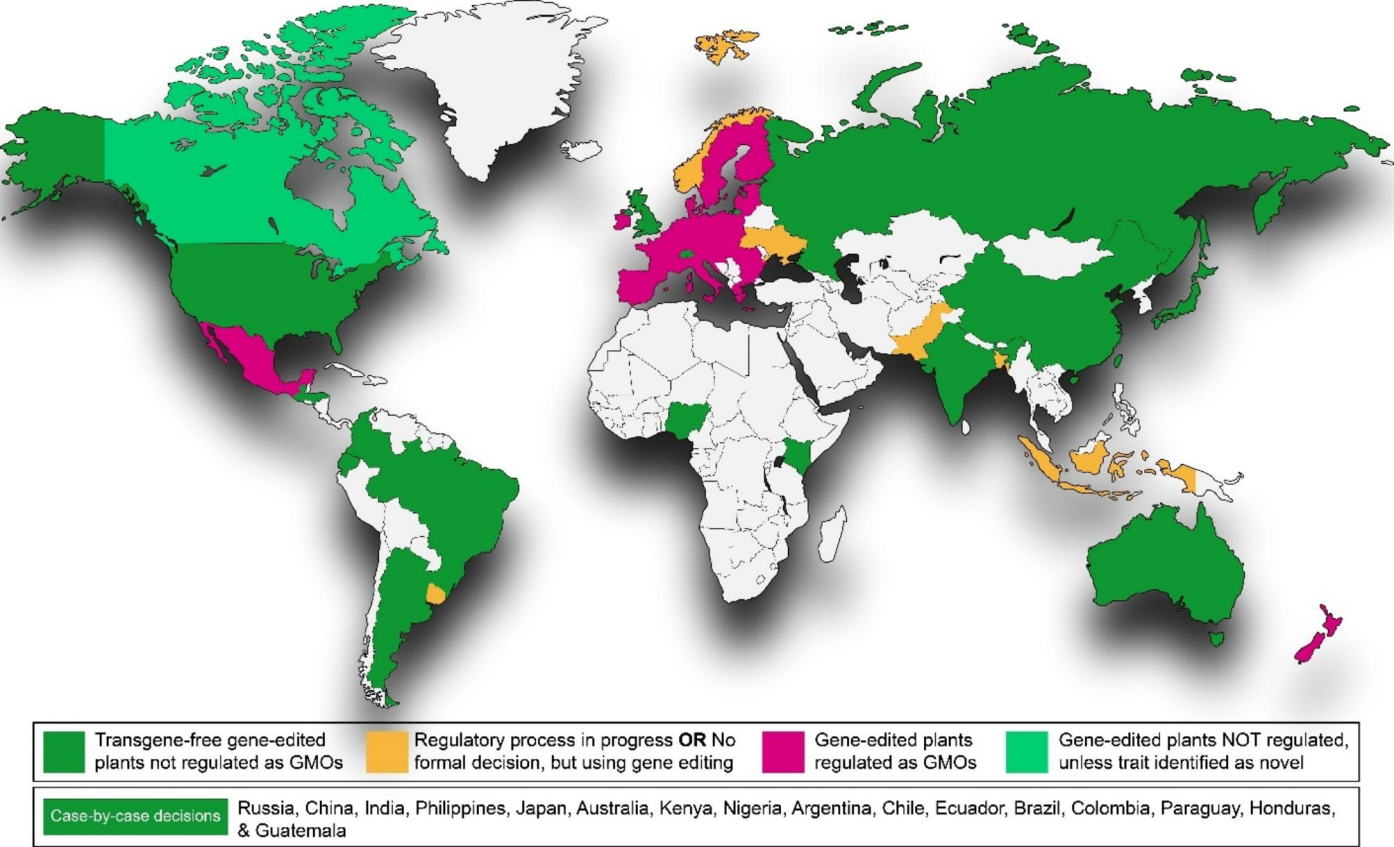
An up-to-date global status of regulation of gene editing or gene-edited plants

In various countries, genetically modified plants that have traces of any foreign DNA are not legal for commercial cultivation. Similarly, whether CRISPR-edited plants should be considered as GMO and pass through the GMO regulatory process or should not be labeled as GMO and pass-on to the next level of field trials and commercialization straight away, is still a question and part of legislative discussions in many countries including European Union (UN). In this regard, some countries have already finished or near to finish their legislative framework and decided the fate of GE crops. Positively, UK has recently joined the countries (i.e., Argentina, Australia, Brazil, Canada, Chile, China, Colombia, India, Israel, Japan, and USA) that have allowed GE tools for crops’ improvements and exempted the CRISPR-edited plants from GMO’s legislations because they possess mutations or genetic changes as similar as they can happen in conventional breeding or natural populations (Caccamo 2023; Schmidt et al. 2020) (Fig. 3).

Additionally, regarding the biosafety concerns, the Ministry of Agriculture and Rural Affairs of China has also recently approved the safety of GE soybean and issued a certificate to the developers for next five years starting from April 2023 (Mallapaty 2022). However, such biosafety approvals should be granted to rice varieties as well to achieve food security globally. In contrast, the GE crops are regulated as GMO in EU and New Zealand. However, Agriculture Ministers from the European Union (EU) have met for the first time to discuss the EU recent proposal on new genomic techniques (NGT), including GE. According to the reports, several EU countries supported proposed suggestions regarding GE technology, as compared to very few who showed their concerns over potential risks. The draft proposal to deregulate NGT, especially GE, was released in early July. While certain traceability requirements would remain in place for all GE crops, the draft foresees that NGT-based plants, which also means GE-plants, that are indistinguishable from ones obtained by conventional breeding should be treated like their conventional counterparts (Union 2023a, b). Likewise, other countries in the world are either working on the proposal and the legislations i.e., Kingdom of Saudi Arabia, Pakistan etc., or still having discussions about the new genomics and precision breeding technologies.

Unlike GMOs, CRISPR-edited plants do not carry any foreign DNA and manipulate the existing gene/s, and it has been proven by many studies (Gu et al. 2021; Li et al. 2020a, b; Yang et al. 2023; Yu et al. 2021). Particularly, CRISPR-edited transgene-free rice plants have been generated and screened through several approaches including genetic segregation, foreign DNA free delivery method or transient expression of CRISPR-Cas systems. Genetic segregation may not affect the plant’s genetic makeup, but it certainly eliminates the vector backbone/selectable marker gene by crossing with wild plants. However, outcrossing itself is a tedious and time-consuming task that could be substituted in future due to some recent developments in CRISPR systems for developing transgene-free plants. For example, grafting and mobile CRISPR by Yang et al. (2023), CDB by Cao et al. (2023) and CRISPR Combo to knock out and fine tune the gene expression simultaneously by Pan et al., (2022) are the versatile breakthroughs that will potentially pave the way towards their smooth application in rice as well as other crops speed and precision breeding programs.

**Fig. 4:**
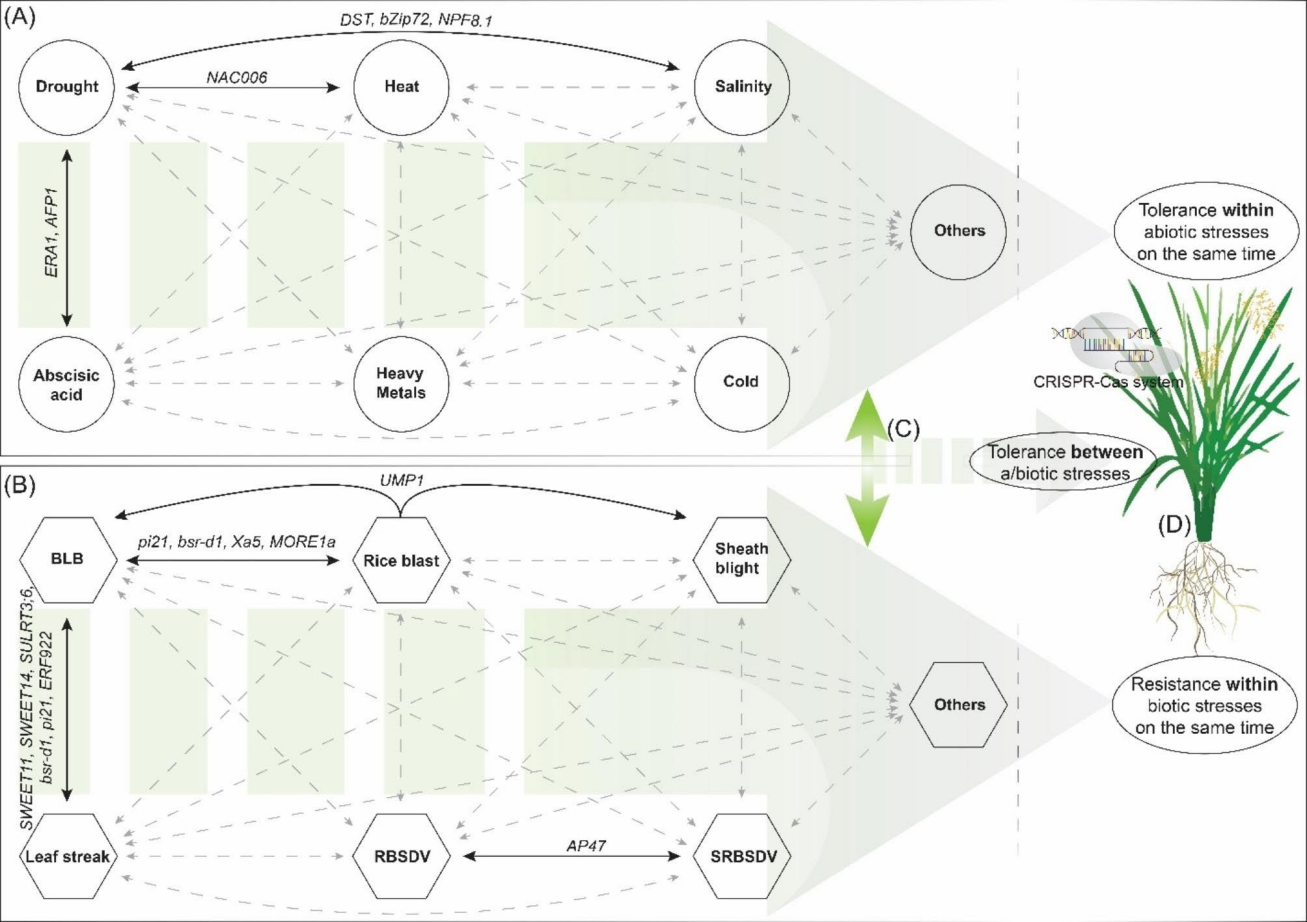
Illustration of proof-of-concept of development of climate-resilient rice lines by manipulating single, double, or multiple genes at the same time and future possibilities. (**A**) Network of abiotic stresses, (**B**) Network of biotic stresses, (**C**) Combined tolerance between abiotic and biotic stresses, (**D**) CRISPR-edited rice line possessing tolerance against multiple stresses at the same time. Solid lines represent the proof-of-concepts of development of tolerance or resistance against multiple stresses at the same time, whereas doted lines show future directions and potential areas in rice gene editing to be explored. BLB, bacterial leaf blight; RBSDV, rice black streak dwarf virus; SRBSDV, southern rice black streak dwarf virus

In conclusion, rice is being consumed as a staple food in half of the world’s countries highlighting the necessity of rice improvement to withstand rapidly changing global climate. Classical plant breeding techniques, along with mutation and transgenic technologies, are struggling hard to meet the global food requirements but cannot be due to their certain limitations and slower pace of action. The rice breeding programs need to be aided through new speed and precision breeding technologies such as CRISPR-Cas based GE systems. Despite of certain limitations, problems and challenges being faced in CRISPR-Cas based GE system, its applications and achievements highlighted in this review show that it has a great potential to revolutionize not only the rice improvement breeding programs but also the future of whole agricultural system to end hunger worldwide. In future, this technology may support the researchers to develop tolerance against multiple abiotic and biotic stresses (both within and between different environmental stresses) simultaneously to save time and resources. In this regard some proof-of-concepts exist in which tolerance or resistance against two or more stresses have been achieved (Tables 1 and 2, and Fig. 4). Here, to conclude our discussion, we hypothesize that development and characterization of CRISPR-edited plants against various stresses at the same time has huge potential that can transform and speed up the future breeding programs (Fig. 4). Additionally, based on the progress and developments being made in CRISPR-Cas systems coupled with rice, we also speculate that they could be one of the key players in bringing the next green revolution.

## Data Availability

Not applicable.

## References

[CR4] Ahmad S, Wei X, Sheng Z (2020). Crispr/cas9 for development of disease resistance in plants: recent progress, limitations and future prospects. Brief Funct Genomics.

[CR1] Ahmad S, Shahzad R, Jamil S et al (2021a) Regulatory aspects, risk assessment, and toxicity associated with rnai and crispr methods Crispr and rnai systems, Elsevier: 687–721

[CR2] Ahmad S, Sheng Z, Jalal RS et al (2021b) Crispr–cas technology towards improvement of abiotic stress tolerance in plants Crispr and rnai systems, Elsevier: 755–772

[CR3] Ahmad S, Tang L, Shahzad R (2021). Crispr-based crop improvements: a way forward to achieve zero hunger. J Agric Food Chem.

[CR6] Alam M, Toriman M, Siwar C The impacts of agricultural supports for climate change adaptation: Farm level assessment study on paddy farmers. Alam, MM, Mohd Ekhwan, Siwar T et al (2011) C., Molla, RI, and Talib, B:178–182

[CR5] Alam M, Kong J, Tao R et al (2022a) Md. Alamin; alotaibi, ss; abdelsalam, nr; xu, j.-h. Crispr/cas9 mediated knockout of the osbhlh024 transcription factor improves salt stress resistance in rice (oryza sativa l.). Plants 2022a, 11, 1184, s Note: MDPI stays neu-tral with regard to jurisdictional claims in &#8230 10.3390/plants11091184 PMC9101608 35567185

[CR7] Alam MS, Kong J, Tao R (2022). Crispr/cas9 mediated knockout of the osbhlh024 transcription factor improves salt stress resistance in rice (oryza sativa l). Plants.

[CR8] Alam MS, Yang Z-K, Li C (2022). Loss-of-function mutations of osbhlh044 transcription factor lead to salinity sensitivity and a greater chalkiness in rice (oryza sativa l). Plant Physiol Biochem.

[CR9] Alfatih A, Wu J, Jan SU (2020). Loss of rice paraquat tolerance 3 confers enhanced resistance to abiotic stresses and increases grain yield in field. Plant Cell Environ.

[CR10] Amit I, Iancu O, Levy-Jurgenson A (2021). Crispector provides accurate estimation of genome editing translocation and off-target activity from comparative ngs data. Nat Commun.

[CR11] Ancha S (2012) Cambodia: Mainstreaming climate resilience into development planning

[CR12] Anjala K, Augustine R (2022). Designing of guide rna constructs for crispr/cas9-mediated editing of rice transcription factor osmads26 for enhancing drought tolerance. J Appl Biology Biotechnol.

[CR13] Aqib Z, Ahmad S, Tabbasum J (2022). Rice grain yield and quality improvement via crispr/cas9 system: an updated review. Notulae Botanicae Horti Agrobotanici Cluj-Napoca.

[CR14] Arra Y, Auguy F, Stiebner M et al (2023) Rice yellow mottle virus resistance by genome editing of the oryza sativa l. ssp. Japonica nucleoporin gene oscpr5. 1 but not oscpr5. 2. bioRxiv:2023.2001. 2013.523077 10.1111/pbi.14266 PMC11022797 38124291

[CR15] Arulganesh T, Kumam Y, Kumar K (2021). Genome editing of elite rice cultivar co51 for bacterial leaf blight resistance. Electron J Plant Breed.

[CR16] Arunrat N, Pumijumnong N, Sereenonchai S (2020). Assessment of climate change impact on rice yield and water footprint of large-scale and individual farming in thailand. Sci Total Environ.

[CR17] Baoxiang W, Yan L, Yifeng W (2021). Osbzip72 is involved in transcriptional gene-regulation pathway of abscisic acid signal transduction by activating rice high-affinity potassium transporter oshkt1; 1. Rice Sci.

[CR18] Beyene G, Chauhan RD, Villmer J (2022). Crispr/cas9-mediated tetra-allelic mutation of the ‘green revolution’semidwarf-1 (sd-1) gene confers lodging resistance in tef (eragrostis tef). Plant Biotechnol J.

[CR19] Biswal AK, Wu T-Y, Urano D (2022). Novel mutant alleles reveal a role of the extra-large g protein in rice grain filling, panicle architecture, plant growth, and disease resistance. Front Plant Sci.

[CR20] Butt H, Rao GS, Sedeek K (2020). Engineering herbicide resistance via prime editing in rice. Plant Biotechnol J.

[CR21] Caccamo M (2023) New precision-breeding law unlocks gene editing in england. Nat Biotechnol :1–2 10.1038/s41587-023-01795-8 37161018

[CR22] Cao X, Xie H, Song M et al (2023) Cut–dip–budding delivery system enables genetic modifications in plants without tissue culture. The Innovation 4(1) 10.1016/j.xinn.2022.100345 PMC9661722 36387605

[CR23] Chang J-D, Gao W, Wang P (2022). Osnramp5 is a major transporter for lead uptake in rice. Environ Sci Technol.

[CR24] Chauhan B (2013). Effect of tillage systems, seeding rates, and herbicides on weed growth and grain yield in dry-seeded rice systems in the philippines. Crop Prot.

[CR26] Chen X, Liu P, Mei L (2021). Xa7, a new executor r gene that confers durable and broad-spectrum resistance to bacterial blight disease in rice. Plant Commun.

[CR25] Chen F, Zhang H, Li H (2023). Ipa1 improves drought tolerance by activating snac1 in rice. BMC Plant Biol.

[CR27] Chen Y, Liu Z, Meng S (2023). Oscerk1 contributes to cupric oxide nanoparticles induced phytotoxicity and basal resistance against blast by regulating the anti-oxidant system in rice. J Fungi.

[CR28] Chung PJ, Chung H, Oh N (2020). Efficiency of recombinant crispr/rcas9-mediated mirna gene editing in rice. Int J Mol Sci.

[CR29] Clemens S (2019). Safer food through plant science: reducing toxic element accumulation in crops. J Exp Bot.

[CR30] Collias D, Beisel CL (2021). Crispr technologies and the search for the pam-free nuclease. Nat Commun.

[CR31] Cui Y, Jiang N, Xu Z (2020). Heterotrimeric g protein are involved in the regulation of multiple agronomic traits and stress tolerance in rice. BMC Plant Biol.

[CR32] Diana PA, Shanthinie A, Arulganesh T (2022). Targeted editing of ossweet13, a bacterial leaf blight susceptible gene in rice using crispr tool. Electron J Plant Breed.

[CR33] Ding Y, Zhang F, Sun F et al (2023) Loss of oshrc function confers blast resistance without yield penalty in rice. Plant biotechnology journal 10.1111/pbi.14061 PMC10363764 37102736

[CR34] Diyang Q, Rui H, Ji L (2023). Peptide transporter osnpf8. 1 contributes to sustainable growth under salt and drought stresses, and grain yield under nitrogen deficiency in rice. Rice Sci.

[CR35] Dong N, Yin W, Liu D (2020). Regulation of brassinosteroid signaling and salt resistance by serk2 and potential utilization for crop improvement in rice. Front Plant Sci.

[CR36] Duy PN, Lan DT, Pham Thu H (2021). Improved bacterial leaf blight disease resistance in the major elite vietnamese rice cultivar tbr225 via editing of the ossweet14 promoter. PLoS ONE.

[CR37] Elkonin LA, Gerashchenkov GA, Borisenko NV et al (2023) Development of sorghum mutants with improved in vitro protein digestibility by crispr/cas9 editing of kafirin genes. The Crop Journal

[CR38] FAO (2023). Temperature change statistics 1961–2022 – global, regional and country trends.

[CR39] Feng P, Guangda W, Peng G (2023). Evaluation of new japonica rice lines with low cadmium accumulation and good quality generated by knocking out osnramp5. Chin J Rice Sci.

[CR40] Fiaz S, Ahmad S, Noor MA (2019). Applications of the crispr/cas9 system for rice grain quality improvement: perspectives and opportunities. Int J Mol Sci.

[CR41] Fiaz S, Khan SA, Ali Noor M et al (2021) Genome engineering for food security. Genome engineering for crop improvement:380–390

[CR42] Field CB, Barros V, Stocker TF et al (2012) Managing the risks of extreme events and disasters to advance climate change adaptation: Special report of the intergovernmental panel on climate change, Cambridge University Press

[CR43] Firdaus RR, Leong Tan M, Rahmat SR (2020). Paddy, rice and food security in malaysia: a review of climate change impacts. Cogent Social Sciences.

[CR44] Gu X, Liu L, Zhang H (2021). Transgene-free genome editing in plants. Front Genome Editing.

[CR45] Guo F, Huang Y, Qi P (2021). Functional analysis of auxin receptor ostir1/osafb family members in rice grain yield, tillering, plant height, root system, germination, and auxinic herbicide resistance. New Phytol.

[CR46] Hamada H, Liu Y, Nagira Y (2018). Biolistic-delivery-based transient crispr/cas9 expression enables in planta genome editing in wheat. Sci Rep.

[CR47] Han X, Chen Z, Li P (2022). Development of novel rice germplasm for salt-tolerance at seedling stage using crispr-cas9. Sustainability.

[CR48] He N, Huang F, Yu M (2022). Analysis of a rice blast resistance gene pita-fuhui2663 and development of selection marker. Sci Rep.

[CR49] Hu X-H, Shen S, Wu J-L et al (2023) A natural allele of proteasome maturation factor improves rice resistance to multiple pathogens. Nat Plants :1–10 10.1038/s41477-022-01327-3 36646829

[CR50] Huang S, Xin S, Xie G (2020). Mutagenesis reveals that the rice osmpt3 gene is an important osmotic regulatory factor. Crop J.

[CR51] Huang Y-Y, Liu X-X, Xie Y (2020). Identification of feronia-like receptor genes involved in rice-magnaporthe oryzae interaction. Phytopathol Res.

[CR52] Huang Y, Han Z, Cheng N (2020). Minor effects of 11 dof family genes contribute to the missing heritability of heading date in rice (oryza sativa l). Front Plant Sci.

[CR53] Hussain B, Ahmad S (2022) Crispr/cas9 for rice crop improvement: recent progress, limitations, and prospects. Mod Techniques Rice Crop Prod :701–717

[CR54] Jagadish S, Murty M, Quick W (2015). Rice responses to rising temperatures–challenges, perspectives and future directions. Plant Cell Environ.

[CR55] Jiang Y, Chai Y, Qiao D (2022). Optimized prime editing efficiently generates glyphosate-resistant rice plants carrying homozygous tap-ivs mutation in epsps. Mol Plant.

[CR56] Jung JH, Li Z, Chen H (2023). Mutation of phytochrome b promotes resistance to sheath blight and saline–alkaline stress via increasing ammonium uptake in rice. Plant J.

[CR57] Kamanga RM, Mbega E, Ndakidemi P (2018). Drought tolerance mechanisms in plants: physiological responses associated with water deficit stress in solanum lycopersicum. Adv Crop Sci Technol.

[CR58] Karavolias NG, Patel D, Seong K et al (2021) Crispr/cas9 knockout of epfl10 reduces stomatal density while maintaining photosynthesis and enhancing water conservation in rice. bioRxiv:2021.2012. 2021.473329

[CR59] Khan I, Khan S, Zhang Y (2021). Crispr-cas technology based genome editing for modification of salinity stress tolerance responses in rice (oryza sativa l). Mol Biol Rep.

[CR61] Kim P, Xue CY, Song HD (2021). Tissue-specific activation of dof11 promotes rice resistance to sheath blight disease and increases grain weight via activation of sweet14. Plant Biotechnol J.

[CR60] Kim CY, Park JY, Choi G (2022). A rice gene encoding glycosyl hydrolase plays contrasting roles in immunity depending on the type of pathogens. Mol Plant Pathol.

[CR62] Kitomi Y, Hanzawa E, Kuya N (2020). Root angle modifications by the dro1 homolog improve rice yields in saline paddy fields. Proc Natl Acad Sci.

[CR63] Kleinstiver BP, Prew MS, Tsai SQ (2015). Engineered crispr-cas9 nucleases with altered pam specificities. Nature.

[CR64] Komatsu A, Ohtake M, Shimatani Z (2020). Production of herbicide-sensitive strain to prevent volunteer rice infestation using a crispr-cas9 cytidine deaminase fusion. Front Plant Sci.

[CR65] Kuang Y, Li S, Ren B (2020). Base-editing-mediated artificial evolution of osals1 in planta to develop novel herbicide-tolerant rice germplasms. Mol Plant.

[CR66] Kumam Y, Rajadurai G, Kumar K et al (2022) Genome editing of indica rice asd16 for imparting resistance against rice tungro disease. J Plant Biochem Biotechnol :1–14

[CR67] Le VT, Kim M-S, Jung Y-J (2022). Research trends and challenges of using crispr/cas9 for improving rice productivity. Agronomy.

[CR68] Lesk C, Rowhani P, Ramankutty N (2016). Influence of extreme weather disasters on global crop production. Nature.

[CR74] Li Y, Ye W, Wang M (2009). Climate change and drought: a risk assessment of crop-yield impacts. Climate Res.

[CR72] Li W, Chern M, Yin J (2019). Recent advances in broad-spectrum resistance to the rice blast disease. Curr Opin Plant Biol.

[CR69] Li C, Li W, Zhou Z (2020). A new rice breeding method: Crispr/cas9 system editing of the xa13 promoter to cultivate transgene-free bacterial blight–resistant rice. Plant Biotechnol J.

[CR71] Li S, Zhang Y, Xia L (2020). Crispr-cas12a enables efficient biallelic gene targeting in rice. Plant Biotechnol J.

[CR73] Li Y, Liao S, Mei P (2021). Oswrky93 dually functions between leaf senescence and in response to biotic stress in rice. Front Plant Sci.

[CR77] Li Z, Sun P, Sun P (2021). Osbc1l1 and osbc1l8 function in stomatal development in rice. Biochem Biophys Res Commun.

[CR70] Li C, Zhou L, Wu B (2022). Improvement of bacterial blight resistance in two conventionally cultivated rice varieties by editing the noncoding region. Cells.

[CR75] Li Z, Huang Q, Lin B (2022). Crispr/cas9-targeted mutagenesis of a representative member of a novel pr10/bet v1-like protein subfamily significantly reduces rice plant height and defense against meloidogyne graminicola. Phytopathol Res.

[CR76] Li Z, Rao MJ, Li J (2022). Crispr/cas9 mutant rice ospmei12 involved in growth, cell wall development, and response to phytohormone and heavy metal stress. Int J Mol Sci.

[CR78] Li Z, Wang B, Luo W (2023). Natural variation of codon repeats in cold11 endows rice with chilling resilience. Sci Adv.

[CR79] Lian T, Huang Y, Xie X (2020). Rice sst variation shapes the rhizosphere bacterial community, conferring tolerance to salt stress through regulating soil metabolites. MSystems.

[CR80] Liang J, Shen Q, Wang L (2021). Rice contains a biosynthetic gene cluster associated with production of the casbane-type diterpenoid phytoalexin ent–10–oxodepressin. New Phytol.

[CR81] Lin D, Kong R, Chen L (2020). Chloroplast development at low temperature requires the pseudouridine synthase gene tcd3 in rice. Sci Rep.

[CR82] Lin QJ, Chu J, Kumar V (2021). Protein phosphatase 2a catalytic subunit pp2a-1 enhances rice resistance to sheath blight disease. Front Genome Editing.

[CR83] Lin W, Kuang H, Bai M et al (2023) Multiplex genome editing targeting soybean with ultra-low anti-nutritive oligosaccharides. The Crop Journal

[CR84] Liu C, Ou S, Mao B (2018). Early selection of bzip73 facilitated adaptation of japonica rice to cold climates. Nat Commun.

[CR86] Liu MH, Kang H, Xu Y (2020). Genome-wide association study identifies an nlr gene that confers partial resistance to magnaporthe oryzae in rice. Plant Biotechnol J.

[CR89] Liu X, Wu D, Shan T (2020). The trihelix transcription factor osgtγ-2 is involved adaption to salt stress in rice. Plant Mol Biol.

[CR90] Liu XH, Lyu YS, Yang W (2020). A membrane-associated nac transcription factor osntl3 is involved in thermotolerance in rice. Plant Biotechnol J.

[CR85] Liu H, Dong S, Li M (2021). The class iii peroxidase gene osprx30, transcriptionally modulated by the at-hook protein osath1, mediates rice bacterial blight–induced ros accumulation. J Integr Plant Biol.

[CR88] Liu X, Song L, Zhang H (2021). Rice ubiquitin-conjugating enzyme osubc26 is essential for immunity to the blast fungus magnaporthe oryzae. Mol Plant Pathol.

[CR91] Liu Y, Luo W, Linghu Q (2021). In planta genome editing in commercial wheat varieties. Front Plant Sci.

[CR87] Liu S, Liu W, Lai J (2022). Osglyi3, a glyoxalase gene expressed in rice seed, contributes to seed longevity and salt stress tolerance. Plant Physiol Biochem.

[CR92] Liying Y, Yuanye Z, Rongtian L (2022). Improvement of herbicide resistance in rice by using crispr/cas9 system. Chin J Rice Sci.

[CR93] Lou D, Lu S, Chen Z (2023). Molecular characterization reveals that ossapk3 improves drought tolerance and grain yield in rice. BMC Plant Biol.

[CR94] Lu G, Wang C, Wang G et al (2020) Knockouts of drought sensitive genes improve rice grain yield under both drought and well-watered field conditions

[CR96] Lu Q, Luo X, Yang X et al (2021) Crispr/cas9-mediated gene editing of vacuolar atpase subunit d mediates phytohormone biosynthesis and virus resistance in rice 10.3389/fpls.2023.1122978 PMC9929465 36818855

[CR95] Lu H, Shen Z, Xu Y et al (2023) Immune mechanism of ethylicin-induced resistance to xanthomonas oryzae pv. Oryzae in rice. Journal of Agricultural and Food Chemistry 10.1021/acs.jafc.2c07385 36591973

[CR97] Lyu Y-S, Cao L-M, Huang W-Q (2022). Disruption of three polyamine uptake transporter genes in rice by crispr/cas9 gene editing confers tolerance to herbicide paraquat. Abiotech.

[CR98] Mallapaty S (2022). China’s approval of gene-edited crops energizes researchers. Nature.

[CR99] Malzahn AA, Tang X, Lee K (2019). Application of crispr-cas12a temperature sensitivity for improved genome editing in rice, maize, and arabidopsis. BMC Biol.

[CR100] MILAzzo J, H ADRE DTHARREAU et al (2022) Crispr/cas9-targeted knockout of rice susceptibility genes osdja2 and oserf104 reveals alternative sources of

[CR101] Mishra R, Joshi RK, Zhao K (2018). Genome editing in rice: recent advances, challenges, and future implications. Front Plant Sci.

[CR102] Monsur MB, Shao G, Lv Y (2020). Base editing: the ever expanding clustered regularly interspaced short palindromic repeats (crispr) tool kit for precise genome editing in plants. Genes.

[CR103] Mushtaq M, Bhat JA, Mir ZA (2018). Crispr/cas approach: a new way of looking at plant-abiotic interactions. J Plant Physiol.

[CR104] Nawaz G, Usman B, Peng H (2020). Knockout of pi21 by crispr/cas9 and itraq-based proteomic analysis of mutants revealed new insights into m. Oryzae resistance in elite rice line. Genes.

[CR105] Ni Z, Cao Y, Jin X (2021). Engineering resistance to bacterial blight and bacterial leaf streak in rice. Rice.

[CR106] Nithya S, Kumam Y, Varanavasiappan S (2020). Targeted mutation in eif4g gene in rice. Electron J Plant Breed.

[CR107] Ogata T, Ishizaki T, Fujita M (2020). Crispr/cas9-targeted mutagenesis of osera1 confers enhanced responses to abscisic acid and drought stress and increased primary root growth under nonstressed conditions in rice. PLoS ONE.

[CR108] Patel SK, Sharma A, Singh GS (2020). Traditional agricultural practices in india: an approach for environmental sustainability and food security. Energy Ecol Environ.

[CR109] Prodhan ZH, Islam SA, Alam MS (2022). Impact of osbadh2 mutations on salt stress response in rice. Plants.

[CR110] Qu M, Essemine J, Li M (2020). Genome-wide association study unravels lrk1 as a dark respiration regulator in rice (oryza sativa l). Int J Mol Sci.

[CR111] Que Z, Lu Q, Liu T et al (2020) The rice annexin gene osann5 is a positive regulator of cold stress tolerance at the seedling stage 10.1002/pld3.539 PMC10628399 37942234

[CR113] Rezvi HUA, Tahjib-Ul–Arif M, Azim MA et al (2022) Rice and food security: Climate change implications and the future prospects for nutritional security. Food and Energy Security:e430

[CR114] Riaz A, Kanwal F, Ahmad I (2022). New hope for genome editing in cultivated grasses: crispr variants and application. Front Genet.

[CR115] Romero FM, Gatica-Arias A (2019). Crispr/cas9: development and application in rice breeding. Rice Sci.

[CR116] Santosh Kumar V, Verma RK, Yadav SK (2020). Crispr-cas9 mediated genome editing of drought and salt tolerance (osdst) gene in indica mega rice cultivar mtu1010. Physiol Mol Biology Plants.

[CR118] Schmidt SM, Belisle M, Frommer WB (2020). The evolving landscape around genome editing in agriculture: many countries have exempted or move to exempt forms of genome editing from gmo regulation of crop plants. EMBO Rep.

[CR117] Schmidt MJ, Gupta A, Bednarski C (2021). Improved crispr genome editing using small highly active and specific engineered rna-guided nucleases. Nat Commun.

[CR119] Shim Y, Seong G, Choi Y et al (2023) Suppression of cuticular wax biosynthesis mediated by rice lov kelch repeat protein 2 supports a negative role in drought stress tolerance. Plant, Cell & Environment 10.1111/pce.14549 36683564

[CR120] Tabassum J, Ahmad S, Hussain B (2021). Applications and potential of genome-editing systems in rice improvement: current and future perspectives. Agronomy.

[CR121] Tang X, Liu G, Zhou J (2018). A large-scale whole-genome sequencing analysis reveals highly specific genome editing by both cas9 and cpf1 (cas12a) nucleases in rice. Genome Biol.

[CR122] Tao H, Shi X, He F (2021). Engineering broad-spectrum disease–resistant rice by editing multiple susceptibility genes. J Integr Plant Biol.

[CR123] Tao J, Bauer DE, Chiarle R (2023). Assessing and advancing the safety of crispr-cas tools: from DNA to rna editing. Nat Commun.

[CR125] Távora FTP (2021) Development of blast resistant rice plants using crispr/cas9 system for genome editing

[CR124] Távora FT, Meunier AC, Vernet A (2022). Crispr/cas9-targeted knockout of rice susceptibility genes osdja2 and oserf104 reveals alternative sources of resistance to pyricularia oryzae. Rice Sci.

[CR126] Teng Y, Lv M, Zhang X (2022). Bear1, a bhlh transcription factor, controls salt response genes to regulate rice salt response. J Plant Biology.

[CR127] Tezuka D, Matsuura H, Saburi W (2021). A ubiquitously expressed udp-glucosyltransferase, ugt74j1, controls basal salicylic acid levels in rice. Plants.

[CR128] Tianshun Z, Dong Y, Ling L (2021). Crispr/cas9-mediatedediting of afp1improves rice stress tolerance. Chin J Rice Sci.

[CR129] Tripathy SP, Majhi PK, Patra B et al (2021) Editing the genome for salt tolerance in rice

[CR130] Tun W, Yoon J, Vo KTX et al (2023) Sucrose preferentially promotes expression of oswrky7 and ospr10a to enhance defense response to blast fungus in rice 10.3389/fpls.2023.1117023 PMC9911862 36778713

[CR131] Turnbull C, Lillemo M, Hvoslef-Eide TA (2021). Global regulation of genetically modified crops amid the gene edited crop boom–a review. Front Plant Sci.

[CR132] Union E (2023a) New techniques in biotechnology

[CR133] Union E (2023b) Regulation of the european parliament and of the council on plants obtained by certain new genomic techniques and their food and feed, and amending regulation (eu) 2017/625 Brussels

[CR134] Usman B, Nawaz G, Zhao N (2020). Precise editing of the ospyl9 gene by rna-guided cas9 nuclease confers enhanced drought tolerance and grain yield in rice (oryza sativa l.) by regulating circadian rhythm and abiotic stress responsive proteins. Int J Mol Sci.

[CR135] Uyeh DD, Asem-Hiablie S, Park T et al (2021) Could japonica rice be an alternative variety for increased global food security and climate change mitigation? Foods 10(8):1869 10.3390/foods10081869 PMC8393797 34441646

[CR136] Wang B, Fang R, Chen F (2020). A novel ccch-type zinc finger protein saw1 activates osga20ox3 to regulate gibberellin homeostasis and anther development in rice. J Integr Plant Biol.

[CR137] Wang B, Zhong Z, Wang X (2020). Knockout of the osnac006 transcription factor causes drought and heat sensitivity in rice. Int J Mol Sci.

[CR139] Wang F, Itai RN, Nozoye T (2020). The bhlh protein osiro3 is critical for plant survival and iron (fe) homeostasis in rice (oryza sativa l.) under fe-deficient conditions. Soil Sci Plant Nutr.

[CR141] Wang K, An W, Liu Y (2020). Disruption of osrhogdi2 by crispr/cas9 technology leads to semi-dwarf in rice. Sheng wu Gong Cheng xue bao = chinese. J Biotechnol.

[CR140] Wang F, Xu Y, Li W (2021). Creating a novel herbicide-tolerance osals allele using crispr/cas9-mediated gene editing. Crop J.

[CR142] Wang W, Ma S, Hu P (2021). Genome editing of rice eif4g loci confers partial resistance to rice black-streaked dwarf virus. Viruses.

[CR143] Wang X, Li J, Li F (2021). Rice potassium transporter oshak8 mediates k + uptake and translocation in response to low k + stress. Front Plant Sci.

[CR145] Wang Z, Chen D, Sun F (2021). Argonaute 2 increases rice susceptibility to rice black-streaked dwarf virus infection by epigenetically regulating hexokinase 1 expression. Mol Plant Pathol.

[CR138] Wang C, Feng X, Yuan Q et al (2022a) Upgrading the genome of an elite japonica rice variety kongyu 131 for lodging resistance improvement. Plant Biotechnology Journal 10.1111/pbi.13963 PMC9884016 36382925

[CR144] Wang X, Ren P, Ji L (2022). Osvde, a xanthophyll cycle key enzyme, mediates abscisic acid biosynthesis and negatively regulates salinity tolerance in rice. Planta.

[CR146] Wang Z, Zhou L, Lan Y (2022). An aspartic protease 47 causes quantitative recessive resistance to rice black-streaked dwarf virus disease and southern rice black–streaked dwarf virus disease. New Phytol.

[CR147] Wei H, Wang X, He Y (2021). Clock component osprr73 positively regulates rice salt tolerance by modulating oshkt2; 1-mediated sodium homeostasis. EMBO J.

[CR148] Wei Z, Abdelrahman M, Gao Y (2021). Engineering broad-spectrum resistance to bacterial blight by crispr-cas9-mediated precise homology directed repair in rice. Mol Plant.

[CR149] Wu Y, Xiao N, Cai Y et al (2023) Crispr/cas9-mediated editing of oshppd 3’-utr confers enhanced resistance to hppd-inhibiting herbicide in rice. Plant communications:100605 10.1016/j.xplc.2023.100605 PMC10504582 37087571

[CR150] Xie Y, Wang Y, Yu X (2022). Sh3p2, an sh3 domain-containing protein that interacts with both pib and avrpib, suppresses effector-triggered, pib-mediated immunity in rice. Mol Plant.

[CR155] Xu Y, Wang F, Chen Z (2020). Intron-targeted gene insertion in rice using crispr/cas9: a case study of the pi-ta gene. Crop J.

[CR151] Xu J, Wang X, Zu H (2021). Molecular dissection of rice phytohormone signaling involved in resistance to a piercing-sucking herbivore. New Phytol.

[CR152] Xu R, Liu X, Li J (2021). Identification of herbicide resistance osacc1 mutations via in planta prime-editing-library screening in rice. Nat Plants.

[CR153] Xu X, Xu Z, Li Z (2021). Increasing resistance to bacterial leaf streak in rice by editing the promoter of susceptibility gene ossulrt3; 6. Plant Biotechnol J.

[CR154] Xu X, Xu Z, Ma W et al (2021d) Tale-triggered and itale-suppressed xa1 resistance to bacterial blight is independent of ostfiiaγ1 or ostfiiaγ5 in rice. Journal of Experimental Botany 10.1093/jxb/erab054 33544818

[CR156] Xu Y, Yan S, Jiang S (2023). Identification of a rice leaf width gene narrow leaf 22 (nal22) through genome-wide association study and gene editing technology. Int J Mol Sci.

[CR157] Yamaguchi K, Yamamoto T, Segami S (2020). Gw2 mutation increases grain width and culm thickness in rice (oryza sativa l). Breed Sci.

[CR158] Yang J, Ji L, Liu S (2021). The cam1-associated ccamk–mkk1/6 cascade positively affects lateral root growth via auxin signaling under salt stress in rice. J Exp Bot.

[CR159] Yang L, Machin F, Wang S et al (2023) Heritable transgene-free genome editing in plants by grafting of wild-type shoots to transgenic donor rootstocks. Nat Biotechnol :1–10 10.1038/s41587-022-01585-8 PMC10344777 36593415

[CR161] Yu Q, Chen L, Zhou W (2020). Rsd1 is essential for stomatal patterning and files in rice. Front Plant Sci.

[CR160] Yu K, Liu Z, Gui H (2021). Highly efficient generation of bacterial leaf blight-resistant and transgene-free rice using a genome editing and multiplexed selection system. BMC Plant Biol.

[CR162] Yudong C, Xiangyi X, Naizhong Y (2021). Auxin regulator osgrf4 simultaneously regulates rice grain shape and blast resistance. Chin J Rice Sci.

[CR163] Yue E, Cao H, Liu B (2020). Osmir535, a potential genetic editing target for drought and salinity stress tolerance in oryza sativa. Plants.

[CR164] Yue E, Rong F, Liu Z (2023). Cadmium induced a non-coding rna microrna535 mediates cd accumulation in rice. J Environ Sci.

[CR165] Zafar K, Khan MZ, Amin I (2020). Precise crispr-cas9 mediated genome editing in super basmati rice for resistance against bacterial blight by targeting the major susceptibility gene. Front Plant Sci.

[CR166] Zafar K, Khan MZ, Amin I et al (2023) Employing template directed crispr-based editing of the osals gene to create herbicide tolerance in basmati rice. AoB PLANTS 10.1093/aobpla/plac059 PMC9977225 36873055

[CR167] Zaman QU, Raza A, Gill RA et al (2023) New possibilities for trait improvement via mobile crispr-rna. Trends in Biotechnology 10.1016/j.tibtech.2023.05.001 37258389

[CR168] Zeng X, Luo Y, Vu NTQ (2020). Crispr/cas9-mediated mutation of ossweet14 in rice cv. Zhonghua11 confers resistance to xanthomonas oryzae pv. Oryzae without yield penalty. BMC Plant Biol.

[CR169] Zeng Y, Wen J, Zhao W (2020). Rational improvement of rice yield and cold tolerance by editing the three genes ospin5b, gs3, and osmyb30 with the crispr–cas9 system. Front Plant Sci.

[CR170] Zhai XY, Chen ZJ, Liu J (2022). Expression of cyp76c6 facilitates isoproturon metabolism and detoxification in rice. J Agric Food Chem.

[CR172] Zhang H, Li L, He Y et al (2020a) Distinct modes of manipulation of rice auxin response factor osarf17 by different plant rna viruses for infection. Proceedings of the National Academy of Sciences 117(16):9112–9121 10.1073/pnas.1918254117 PMC7183187 32253321

[CR178] Zhang Y, Wang X, Luo Y (2020). Osaba8ox2, an aba catabolic gene, suppresses root elongation of rice seedlings and contributes to drought response. Crop J.

[CR175] Zhang R, Chen S, Meng X (2021). Generating broad-spectrum tolerance to als-inhibiting herbicides in rice by base editing. Sci China Life Sci.

[CR177] Zhang Y, Ren Q, Tang X (2021). Expanding the scope of plant genome engineering with cas12a orthologs and highly multiplexable editing systems. Nat Commun.

[CR173] Zhang M, Zhao R, Huang K (2022). The oswrky63–oswrky76–osdreb1b module regulates chilling tolerance in rice. Plant J.

[CR176] Zhang X, Liu D, Gao D (2022). Cytokinin confers brown planthopper resistance by elevating jasmonic acid pathway in rice. Int J Mol Sci.

[CR171] Zhang A, He H, Li Y (2023). Mads-box subfamily gene gmap3 from glycine max regulates early flowering and flower development. Int J Mol Sci.

[CR174] Zhang M, Zhao R, Huang K et al (2023b) Oswrky76 positively regulates drought stress via osbhlh148-mediated jasmonate signaling in rice 10.3389/fpls.2023.1168723 PMC10113545 37089644

[CR179] Zhao F-J, Ma Y, Zhu Y-G (2015). Soil contamination in china: current status and mitigation strategies. Environ Sci Technol.

[CR180] Zhao W, Xiao W, Sun J (2022). An integration of microrna and transcriptome sequencing analysis reveal regulatory roles of mirnas in response to chilling stress in wild rice. Plants.

[CR181] Zhou Y, Xu S, Jiang N (2022). Engineering of rice varieties with enhanced resistances to both blast and bacterial blight diseases via crispr/cas9. Plant Biotechnol J.

[CR182] Zhu Z, Yin J, Chern M (2020). New insights into bsr-d1–mediated broad–spectrum resistance to rice blast. Mol Plant Pathol.

